# Incorporating Polygenic Risk Scores and Nongenetic Risk Factors for Breast Cancer Risk Prediction Among Asian Women

**DOI:** 10.1001/jamanetworkopen.2021.49030

**Published:** 2022-03-21

**Authors:** Yaohua Yang, Ran Tao, Xiang Shu, Qiuyin Cai, Wanqing Wen, Kai Gu, Yu-Tang Gao, Ying Zheng, Sun-Seog Kweon, Min-Ho Shin, Ji-Yeob Choi, Eun-Sook Lee, Sun-Young Kong, Boyoung Park, Min Ho Park, Guochong Jia, Bingshan Li, Daehee Kang, Xiao-Ou Shu, Jirong Long, Wei Zheng

**Affiliations:** 1 Division of Epidemiology, Department of Medicine, Vanderbilt Epidemiology Center, Vanderbilt-Ingram Cancer Center, Vanderbilt University Medical Center, Nashville, Tennessee; 2 Department of Biostatistics, Vanderbilt University Medical Center, Nashville, Tennessee; 3 Department of Epidemiology & Biostatistics, Memorial Sloan Kettering Cancer Center, New York, New York; 4 Shanghai Municipal Center for Disease Control and Prevention, Shanghai Institutes of Preventive Medicine, Shanghai, China; 5 State Key Laboratory of Oncogene and Related Genes and Department of Epidemiology, Shanghai Cancer Institute, Renji Hospital, Shanghai Jiaotong University School of Medicine, Shanghai, China; 6 Department of Cancer Prevention, Fudan University Shanghai Cancer Center, Shanghai, China; 7 Department of Preventive Medicine, Chonnam National University Medical School, Hwasun, South Korea; 8 Jeonnam Regional Cancer Center, Chonnam National University Hwasun Hospital, Hwasun, South Korea; 9 Department of Biomedical Sciences, Seoul National University College of Medicine, Seoul, South Korea; 10 Department of Preventive Medicine, Seoul National University College of Medicine, Seoul, South Korea; 11 Cancer Research Institute, Seoul National University College of Medicine, Seoul, South Korea; 12 National Cancer Center Graduate School of Cancer Science and Policy, Goyang, South Korea; 13 Hospital, National Cancer Center, Goyang, South Korea; 14 Research Institute, National Cancer Center, Goyang, South Korea; 15 Department of Preventive Medicine, Hanyang University College of Medicine, Seoul, South Korea; 16 Department of Surgery, Chonnam National University Medical School & Hospital, Hwasun, South Korea; 17 Department of Molecular Physiology & Biophysics, Vanderbilt Genetics Institute, Vanderbilt University, Nashville, Tennessee; 18 Department of Biomedical Sciences, Seoul National University Graduate School, Seoul, South Korea; 19 Institute of Environmental Medicine, Seoul National University Medical Research Center, Seoul, South Korea

## Abstract

**Importance:**

Polygenic risk scores (PRSs) have shown promise in breast cancer risk prediction; however, limited studies have been conducted among Asian women.

**Objective:**

To develop breast cancer risk prediction models for Asian women incorporating PRSs and nongenetic risk factors.

**Design, Setting, and Participants:**

This diagnostic study included women of Asian ancestry from the Asia Breast Cancer Consortium. PRSs were developed using data from genomewide association studies (GWASs) of breast cancer conducted among 123 041 women with Asian ancestry (including 18 650 women with breast cancer) using 3 approaches: (1) reported PRS for women with European ancestry; (2) breast cancer–associated single-nucleotide variations (SNVs) identified by fine-mapping of GWAS-identified risk loci; and (3) genomewide risk prediction algorithms. A nongenetic risk score (NGRS) was built, including 7 well-established nongenetic risk factors, using data of 416 case participants and 1558 control participants from a prospective cohort study. PRSs were initially validated in an independent data set including 1426 case participants and 1323 control participants and further evaluated, along with the NGRS, in the second data set including 368 case participants and 736 control participants nested within a prospective cohort study.

**Main Outcomes and Measures:**

Logistic regression was used to examine associations of risk scores with breast cancer risk to estimate odds ratios (ORs) with 95% CIs and area under the receiver operating characteristic curve (AUC).

**Results:**

A total of 126 894 women of Asian ancestry were included; 20 444 (16.1%) had breast cancer. The mean (SD) age ranged from 49.1 (10.8) to 54.4 (10.4) years for case participants and 50.6 (9.5) to 54.0 (7.4) years for control participants among studies that provided demographic characteristics. In the prospective cohort, a PRS with 111 SNVs developed using the fine-mapping approach (PRS111) showed a prediction performance comparable with a genomewide PRS that included more than 855 000 SNVs. The OR per SD increase of PRS111 score was 1.67 (95% CI, 1.46-1.92), with an AUC of 0.639 (95% CI, 0.604-0.674). The NGRS had a limited predictive ability (AUC, 0.565; 95% CI, 0.529-0.601). Compared with the average risk group (40th-60th percentile), women in the top 5% of PRS111 and NGRS were at a 3.84-fold (95% CI, 2.30-6.46) and 2.10-fold (95% CI, 1.22-3.62) higher risk of breast cancer, respectively. The prediction model including both PRS111 and NGRS achieved the highest prediction accuracy (AUC, 0.648; 95% CI, 0.613-0.682).

**Conclusions and Relevance:**

In this study, PRSs derived using breast cancer risk–associated SNVs had similar predictive performance in Asian and European women. Including nongenetic risk factors in models further improved prediction accuracy. These findings support the utility of these models in developing personalized screening and prevention strategies.

## Key Points

**Question:**

How well do breast cancer risk prediction models that incorporate polygenic risk scores (PRSs) and nongenetic risk factors perform for Asian women?

**Findings:**

In this diagnostic study of 126 894 women, a PRS including 111 genetic variants was developed and tested using data from a prospective cohort study. The PRS was significantly associated with breast cancer risk, and adding 7 nongenetic risk factors improved the model’s accuracy.

**Meaning:**

These findings support the utility of prediction models in identifying Asian women with high risk of breast cancer.

## Teaser

This diagnostic study develops and tests breast cancer risk prediction models for Asian women, incorporating polygenic risk scores (PRSs) and nongenetic risk factors.

## Introduction

Breast cancer is the most commonly diagnosed malignant neoplasm among women worldwide.^1^ The incident rate of breast cancer has been increasing substantially in multiple Asian countries, many of which do not have a population-based breast cancer screening program, leading to delayed cancer diagnoses and poor survival rates.^2^ Because of the differences in breast cancer risk, screening programs currently implemented in the US and European countries may not be appropriate for Asian countries. Hence, a cost-efficient, population-specific breast cancer screening strategy for Asian women is needed.

In 2006, we established the Asia Breast Cancer Consortium (ABCC) to identify genetic risk variants, including single nucleotide variants (SNVs), associated with breast cancer risk through genomewide association studies (GWASs). Approximately 50 risk loci were identified using Asian data alone or in combination with data from European descendants.^3,4,5,6,7,8,9,10^ However, most breast cancer risk loci were identified in GWASs conducted in European descendants.^11^ Multiple studies have attempted to aggregate the associations of risk SNVs identified by GWAS as polygenic risk scores (PRSs) to stratify women into different breast cancer risk groups.^12,13,14,15,16^ Most PRSs for breast cancer were established specifically in women of European ancestry, and among them, a 313-SNV PRS showed the highest predictive ability, with an area under the receiver operating characteristic curve (AUC) of 0.630 to 0.642.^12^ To our knowledge, few studies of breast cancer PRSs for Asian women have been conducted, and limited prediction accuracy was observed.^17,18,19,20,21^ A recent study showed that the 313-SNV PRS performed better than PRSs derived from Asian data.^21^ However, in that study, the Asian-specific PRSs were derived using a limited number of SNVs.

Nongenetic factors are also associated with breast cancer risk.^22^ Several studies have explored the potential of incorporating PRSs and nongenetic risk factors in improving predictive accuracy.^16,22,23,24,25^ Among them, a recent study among European women revealed that the 313-SNV PRS was more predictive than a model including 16 nongenetic risk factors, and the best risk stratification performance was achieved when PRS and nongenetic factors were combined.^22^ However, to our knowledge, similar studies have rarely been carried out among Asian women. In the present study, we aimed to develop and validate PRSs for Asian women using data from the largest GWAS of breast cancer conducted among Asian women that we are aware of and to further evaluate the performance of risk prediction models, including both PRSs and nongenetic risk factors.

## Methods

### Study Participants

In this diagnostic study, the PRS development data sets included GWAS data of 20 076 women with breast cancer and 105 714 control participants of Asian ancestry from the ABCC (Table 1). Detailed information on the ABCC is described elsewhere.^10^ We divided these data sets to a training set (18 650 case participants and 104 391 control participants) for PRS derivation and a validation set (1426 case participants and 1323 control participants) for prediction performance evaluation (eMethods in the Supplement). For each PRS development approach, the most predictive PRS in our validation set was further evaluated in an independent case-control study nested within a prospective cohort study, comprising 368 case participants and 736 control participants, individually matched by age (<5 years). Included in the nested case-control study were participants from the Shanghai Women’s Health Study (SWHS), and none of them had a diagnosis of any cancers at the time of enrollment (eMethods in the Supplement).^10,26^ All studies involved in the current analyses have been approved by their respective institutional review boards. All participants provided informed consent prior to study inclusion. This study followed the Transparent Reporting of a Multivariable Prediction Model for Individual Prognosis or Diagnosis (TRIPOD) reporting guideline.

**Table 1.** Summary of Participating Studies Included in the Current Study

Study	Case participants, No.	Control participants, No.	Age at enrollment, mean (SD), y^**a**^	
			Case participants	Control participants
PRS training and testing				
Training set				
SBCGS	5384	6347	52.8 (9.3)	52.1 (9.2)
HCES-Br	274	273	49.1 (10.8)	54.0 (7.4)
KPOP	963	921	NA	NA
BBJ2	5552	89 731	NA	NA
SeBCS	2246	2052	NA	NA
BCAC-Asians	4231	5067	54.4 (10.4)	53.8 (10.0)
Validation set				
SBCGS	1426	1323	50.1 (11.3)	50.6 (9.5)
Subtotal	20 076	105 714	NA	NA
Prospective study				
SWHS	368	736	52.1 (8.7)	51.6 (9.5)

Abbreviations: BBJ2, The Biobank Japan Project 2; BCAC, Breast Cancer Association Consortium; HCES-Br, Hwasun Cancer Epidemiology Study–Breast; KPOP, Korea Precision Oncology Program; NA, not applicable; PRS, polygenic risk score; SBCGS, Shanghai Breast Cancer Genetic Study; SeBCS, Seoul Breast Cancer Study; SWHS, Shanghai Women’s Health Study.

^a^ Individual level data were not available for KPOP, BBJ2, and SeBCS.

### Genetic Data

Detailed descriptions of genetic data are provided in our recent publication^10^ and in the eMethods in the Supplement. Genotyping was conducted using several platforms, and genotyping data imputation was performed separately by study (eTable 1 in the Supplement). GWAS was conducted within each study and/or substudy, and association results were combined via fixed-effects meta-analyses.

### PRS Development

We applied 3 approaches to develop PRSs, as described briefly here and in detail in the eMethods in the Supplement. PRSs were calculated using the following formula: 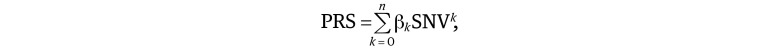
in which SNV*_k_* and β*_k_* represent the allelic dosage and corresponding weight of SNV *k*, and *n* is the number of SNVs used.

### Reported PRS for Women With European Ancestry

To our knowledge, the best breast cancer PRS to date was the 313-SNV PRS among women with European ancestry.^12^ Most recently, this PRS was updated by adding 17 novel breast cancer–susceptibility SNVs.^11^ Of these 330 SNVs, 263 could be found in our validation set and prospective test set, and 3 PRSs (PRS263-Asian, PRS263-European, and PRS263-meta) were derived using weights of these SNVs from data from women with European ancestry included in the Breast Cancer Association Consortium (BCAC-European),^11^ data from women with Asian ancestry in our training set (Table 1), and meta-analyses of these 2 data sets, respectively.

### PRSs Based on SNVs Selected From Fine-Mapping of GWAS-Identified Risk Loci

As shown in the eFigure in the Supplement, for each of the 238 independent susceptibility loci for breast cancer,^3,4,5,6,7,8,9,10,27^ fine-mapping analyses were performed using data from our training set to identify SNVs that were independently associated with breast cancer using Genomewide Complex Trait Analysis–conditional and joint association analysis (GCTA-COJO) version 1.93.2.^28,29^ Within each locus, a COJO threshold of *P* < 1 × 10^−5^ was used to identify independently associated SNVs and reestimate weights of their association with breast cancer risk. Some loci were ineligible for fine-mapping because no SNVs within them had an association with breast cancer risk at *P* < 10^−5^ in our training set. Based on fine-mapping, 3 PRSs were derived using (1) all SNVs selected from fine-mapping; (2) SNVs selected by fine-mapping and showing consistent association directions with *P* < .05 in the BCAC-European data; and (3) SNVs in the second PRS and lead SNVs from loci that were ineligible for fine-mapping but showed *P* < .05 in our training set (eFigure in the Supplement). We repeated the fine-mapping analyses at COJO thresholds of *P* < 1 × 10^−3^ and *P* < 1 × 10^−4^ to identify SNVs and used them to construct 3 sets of PRSs for each threshold following the same steps described previously.

### PRSs Based on Genomewide Risk Prediction Algorithms

LDpred (version 1.0.11), LDpred2 (version 1.4.4), and PRS-CSx (July 29, 2021, release) were used to derive PRSs using data from the training set. Details of these algorithms can be obtained elsewhere.^30,31,32^ Of a total of 5 947 015 SNVs, indels and ambiguous SNVs were excluded by LDpred, and weights of the associations of the remaining 4 487 284 SNVs with breast cancer were reevaluated. Both LDpred2 and PRS-CSx recommend using SNVs included in HapMap 3; thus, of the 5 947 015 SNVs, weights of only 855 680 HapMap 3 SNVs were reestimated (eMethods in the Supplement).

### Models Incorporating PRSs and Nongenetic Risk Factors

Established nongenetic breast cancer risk factors included body mass index (BMI; calculated as weight in kilograms divided by height in meters squared), waist-to-hip ratio (WHR), benign breast disease, age at menarche, age at first live birth, and family history of breast cancer. The interaction between BMI and menopause status was included in the model, as BMI shows a different association with breast cancer risk by menopausal status.^17^ Data from 1974 women (416 case participants and 1558 control participants) from the SWHS, but independent from those in the prospective test set, were used to estimate the weights of the association of these 7 nongenetic factors and the interaction term with breast cancer risk (eTable 2 in the Supplement). Missing data were only found for age at menarche (1 and 2 participants in the training and test sets, respectively) and age at first live birth (72 and 40 participants in the training and test sets, respectively), which were imputed using the R function mice::mice (R version 3.6.0 [R Project for Statistical Computing]). A logistic regression model was fitted with case or control status as the outcome and these 8 factors set as predictors. Weights estimated from this model were then used to construct a nongenetic risk score (NGRS) for each participant using the following formula: 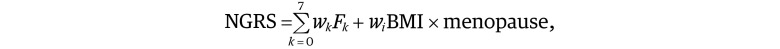
where *F_k_* and *w_k_* are the value and corresponding weight of factor *k*, and *w_i_* is the weight of the interaction term between BMI and menopause status.

### Statistical Analysis

#### Prediction Performance Evaluation

For the PRS showing the highest prediction accuracy in our prospective test set and the reported European PRS,^12^ an integrated risk prediction model (IRPM) was built including a PRS and the NGRS to predict breast cancer risk (breast cancer risk equals approximately PRS + NGRS). Logistic regression was used to evaluate ORs and 95% CIs per SD increase in these risk scores. Prediction performance was measured by AUCs and 95% CIs using the R function pROC:roc.^33^

#### Absolute Risk of Developing Breast Cancer According to PRS Percentiles

We estimated the 10-year absolute risk of developing breast cancer using the most predictive PRS in our prospective test set and the reported European PRS.^12^ A total of 10 207 Chinese women (5087 case participants and 5120 control participants) from the Shanghai Breast Cancer Genetic Study and BCAC-Asians (eTable 1 in the Supplement) were included in this analysis. Logistic regression was used to estimate breast cancer ORs of different PRS percentile groups compared with the middle quintile (40th to 60th percentile) group. Then 10-year absolute risks were calculated using these ORs and the incidence and mortality rates of breast cancer in Shanghai in 2017 following the strategy described previously.^17^ ORs with a 2-tailed *P* < .05 were considered statistically significant.

## Results

A total of 126 894 women of Asian ancestry from the ABCC, including 20 444 (16.1%) with breast cancer and 106 450 control participants, were included in this study (Table 1). The ABCC data sets were divided into a PRS derivation set (18 650 case participants and 104 391 control participants), a validation set (1426 case participants and 1323 control participants), and a prospective test set (368 case participants and 736 control participants). The mean (SD) age ranged from 49.1 (10.8) to 54.4 (10.4) years for case participants and 50.6 (9.5) to 54.0 (7.4) years for control participants among studies that provided demographic characteristics data to the consortium.

### Prediction Performance of PRSs

In general, all PRSs had a slightly higher mean value among case participants than among control participants, while the SD value was similar between both groups, in both the case-control validation set and the prospective test set (eTable 3 in the Supplement). The 3 PRSs derived based on the reported European PRS had similar prediction performance in our validation set (Table 2 and eTable 3 and eTable 4 in the Supplement). However, in our prospective test set, PRS263-meta had the best prediction accuracy (AUC, 0.626; 95% CI, 0.592-0.661) (Table 2). The OR of breast cancer per SD increase of PRS263-meta score was 1.63 (95% CI, 1.43-1.87).

**Table 2.** Associations of PRSs With Breast Cancer Risk in the Validation Set and Prospective Test Set, the Asia Breast Cancer Consortium

PRS development methods	Validation set (1426 case participants vs 1323 control participants)			Prospective test set (368 case participants vs 736 control participants)		
	OR (95% CI)^a^	AUC (95% CI)	*P* value^**a**^	OR (95% CI)^a^	AUC (95% CI)	*P* value^**a**^
Published European PRS^b^						
PRS263-European	1.42 (1.31 to 1.53)	0.597 (0.575 to 0.618)	2.47 × 10^−18^	1.62 (1.42 to 1.85)	0.625 (0.590 to 0.659)	2.71 × 10^−12^
PRS263-Asian	1.44 (1.33 to 1.56)	0.601 (0.580 to 0.622)	5.47 × 10^−20^	1.58 (1.38 to 1.80)	0.616 (0.582 to 0.651)	1.41 × 10^−11^
PRS263-meta	1.44 (1.33 to 1.55)	0.600 (0.579 to 0.621)	1.54 × 10^−19^	1.63 (1.43 to 1.87)	0.626 (0.592 to 0.661)	1.25 × 10^−12^
Fine-mapping^c^						
PRS111, with COJO *P* < 1 × 10^−5^	1.45 (1.34 to 1.57)	0.603 (0.582 to 0.624)	2.72 × 10^−20^	1.67 (1.46 to 1.92)	0.639 (0.604 to 0.674)	1.28 × 10^−13^
PRS112, with COJO *P* < 1 × 10^−4^	1.42 (1.31 to 1.53)	0.597 (0.575 to 0.618)	1.38 × 10^−18^	1.63 (1.42 to 1.87)	0.632 (0.597 to 0.667)	1.70 × 10^−12^
PRS135, with COJO *P* < 1 × 10^−3^	1.38 (1.28 to 1.49)	0.592 (0.571 to 0.613)	3.30 × 10^−16^	1.54 (1.35 to 1.76)	0.619 (0.584 to 0.655)	1.55 × 10^−10^
Genomewide risk prediction algorithms^d^						
LDpred, with 4 487 284 SNVs	1.44 (1.34 to 1.56)	0.600 (0.579 to 0.621)	4.96 × 10^−20^	1.52 (1.34 to 1.74)	0.616 (0.581 to 0.651)	4.08 × 10^−10^
LDpred2, with 855 680 SNVs	1.40 (1.29 to 1.51)	0.591 (0.570 to 0.612)	4.77 × 10^−17^	1.51 (1.33 to 1.72)	0.612 (0.577 to 0.648)	7.47 × 10^−10^
PRS-CSx, with 855 680 SNVs	1.51 (1.39 to 1.63)	0.613 (0.592 to 0.634)	3.03 × 10^−24^	1.70 (1.49 to 1.95)	0.642 (0.608 to 0.676)	1.37 × 10^−14^

Abbreviations: AUC, area under the receiver operating characteristic curve; COJO, conditional and joint association; OR, odds ratio; PRS, polygenic risk score; SNVs, single-nucleotide variants.

^a^ OR per SD increase in PRS scores; *P* values were estimated using logistic regression.

^b^ Of the 330 SNVs included in the European-ancestry PRS reported by Zhang et al,^11^ data on 263 SNVs were available in our validation and prospective test sets and thus included in this analysis. These PRSs were developed using weights from Breast Cancer Association Consortium–European data only (PRS263-European), Asian data only (PRS263-Asian), and meta-analyses of these 2 data sets (PRS263-meta), respectively.

^c^ Developed using SNVs selected from fine-mapping of Asian data and showing consistent association directions in Breast Cancer Association Consortium–European data with *P* < .05. All weights were derived using Asian data.

^d^ For each algorithm, only the most predictive PRS in the validation set is presented. Weights of SNVs from our training set were estimated using each algorithm.

**Figure 1.**
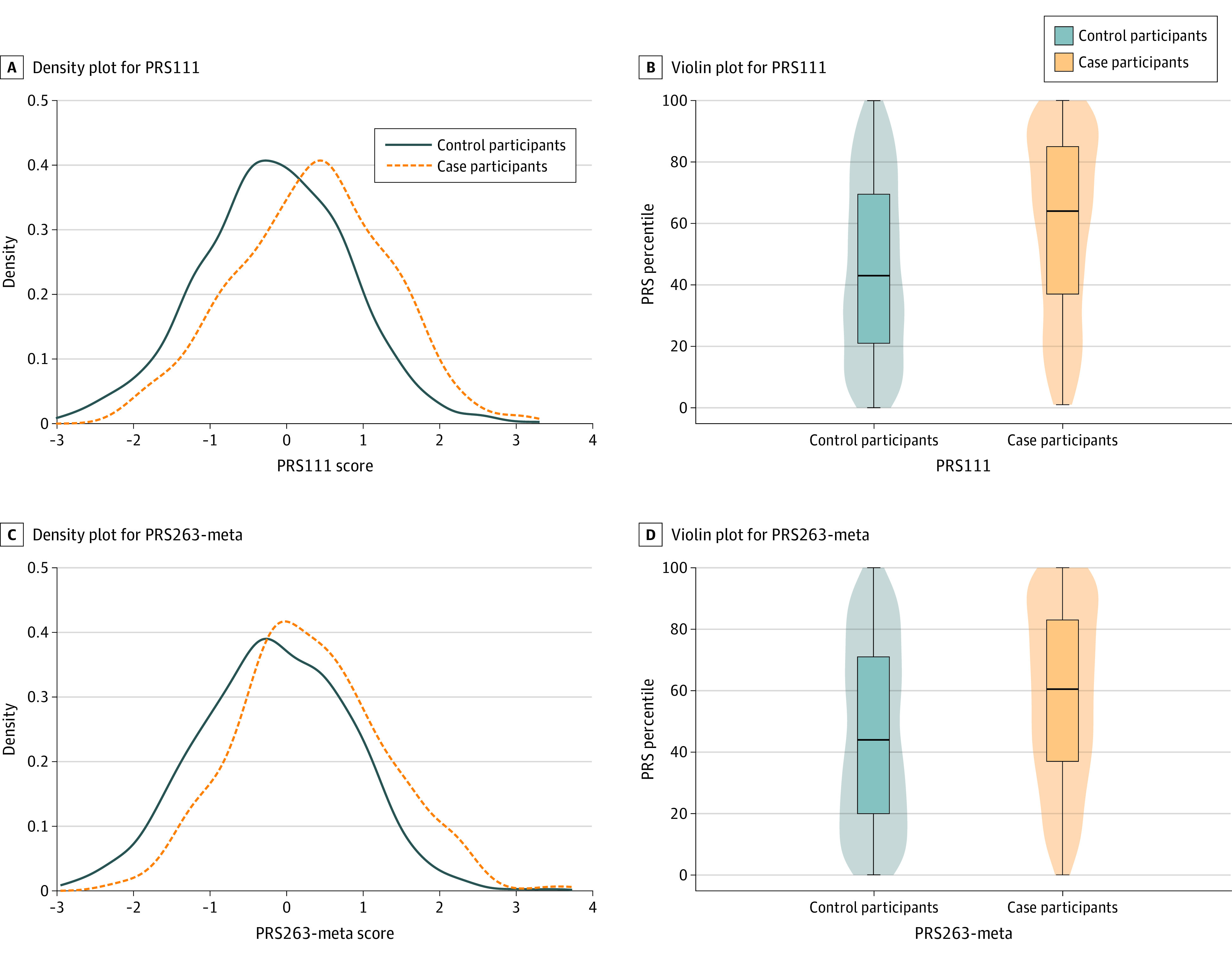
Distributions of Scores on Standardized Polygenic Risk Scores (PRSs) Among Patients With and Without Breast Cancer in the Prospective Test Set The PRS was standardized by subtracting the mean and dividing by the standard deviation. B and D, The upper edge, center line, and lower edge of the box represent the first, second, and third quartiles, respectively, of PRS percentile. The whiskers indicate the full range of the data. PRS111 indicates the PRS using 111 single-nucleotide variants; PRS263-meta, the PRS based on a meta-analysis of European and Asian data.

Using the fine-mapping approach, 3 PRSs were developed at each fine-mapping threshold (eTable 3 in the Supplement), and among them, PRS111 showed the strongest association with breast cancer risk and highest prediction performance in both validation and prospective test sets (Table 2). This PRS was developed using 57 SNVs selected by fine-mapping and showing consistent association directions with *P* < .05 in the BCAC-European data,^11^ plus 54 lead SNVs in GWAS loci with *P* < .05 in our training set (eFigure and eTable 5 in the Supplement). The OR for breast cancer per SD increase in PRS111 score was 1.45 (95% CI, 1.34-1.57) in our validation set and 1.67 (95% CI, 1.46-1.92) in our prospective test set, with AUCs of 0.603 (95% CI, 0.582-0.624) and 0.639 (95% CI, 0.604-0.674), respectively (Table 2). Compared with the average risk group (40th-60th percentile), women in the top 5% of PRS111 were at 3.84-fold (95% CI, 2.30-6.46) increased risk of breast cancer. Among participants younger than 60 years, the OR of the association between PRS111 score and breast cancer risk increased with age; however, this reversed among women older than 60 years (eTable 6 in the Supplement). No significant interaction between PRS111 and age was observed. For both PRS111 and PRS263-meta, distribution curves for case participants were shifted to the right compared with those for control participants, and the overlap was less for PRS111 than PRS263-meta (Figure 1)_._ The difference in median percentile between case and control participants was higher for PRS111 (64 [37-85] vs 43 [21-69]) compared with PRS263-meta (60 [37-83] vs 44 [20-71]) (Figure 1).

Among the most predictive PRS derived by each genomewide risk prediction algorithm, the PRS derived from PRS-CSx was the best-performing PRS in both the validation and the prospective test set, with AUCs of 0.613 (95% CI, 0.592-0.634) and 0.642 (95% CI, 0.608-0.676), respectively (Table 2 and eTable 3 in the Supplement). The OR for breast cancer per SD increase of this PRS in these 3 data sets was 1.51 (95% CI, 1.39-1.63) and 1.70 (95% CI, 1.49-1.95), respectively. Although the PRS derived from PRS-CSx performed slightly better than PRS111 (AUCs, 0.642 vs 0.639), we chose PRS111 for downstream analyses because it used fewer SNVs than the PRS derived from PRS-CSx (111 vs 855 680 SNVs) but had almost equal predictive ability.

### Prediction Performance of NGRS and IRPMs

In the training set for NGRS, compared with control participants, case participants, on average, had a younger age at menarche, older age at first live birth, and higher BMI (eTable 2 in the Supplement). More case than control participants were postmenopausal and had a family history of breast cancer or a prior diagnosis of benign breast diseases. Although not all statistically significant, these findings were consistent with those that have been reported previously. Similar patterns of the associations were found in the prospective test set, although not all associations were statistically significant. Compared with the training set, women included in the prospective test set tended to be older, were more likely to be postmenopausal (576 of 1104 [52.2%] vs 908 of 1974 [46.0%]), and had a lower prevalence of prior benign breast disease diagnosis (174 of 1104 [15.8%] vs 395 of 1974 [20.0%]).

In our prospective test set, the NGRS was associated with breast cancer risk with an OR per SD increase of 1.29 (95% CI, 1.14-1.46) and an AUC of 0.565 (95% CI, 0.529-0.601) (Table 3). Incorporating the NGRS with PRS111 or PRS263-meta, we established 2 models, IRPM111 and IRPM263-meta, respectively. Within both models, the PRS and the NGRS were independently associated with breast cancer, although the PRS showed a stronger association than the NGRS (Table 3). IRPM111 showed a better prediction accuracy (AUC, 0.648; 95% CI, 0.613-0.682) than IRPM263-meta (AUC, 0.632; 95% CI, 0.597-0.666) (Table 3). In the prospective test set, family history was associated with breast cancer risk independently of other nongenetic factors (OR, 2.17; 95% CI, 1.10-4.28), and this association was slightly attenuated after adjusting for PRS111 (adjusted OR, 2.11; 95% CI, 1.06-4.22).

**Table 3.** Performance of Risk Scores in the Prospective Test Set

Model	AUC (95% CI)	Predictor	OR (95% CI)^**a**^	*P* value^**a**^
NGRS^b^	0.565 (0.529-0.601)	NGRS^b^	1.29 (1.14-1.46)	6.36 × 10^−5^
PRS111^c^	0.639 (0.604-0.674)	PRS111^c^	1.67 (1.46-1.92)	1.28 × 10^−13^
PRS263-meta^d^	0.626 (0.592-0.661)	PRS263-meta^d^	1.63 (1.43-1.87)	1.25 × 10^−12^
IRPM111^c^	0.648 (0.613-0.682)	PRS111^c^	1.66 (1.46-1.91)	2.14 × 10^−13^
		NGRS^b^	1.17 (1.03-1.33)	.02
IRPM263-meta^d^	0.632 (0.597-0.666)	PRS263-meta^d^	1.62 (1.42-1.86)	2.91 × 10^−12^
		NGRS^b^	1.16 (1.02-1.32)	.02

Abbreviations: AUC, area under the receiver operating characteristic curve; IRPM, integrated risk prediction model; NGRS, nongenetic risk score; OR, odds ratio; PRS, polygenic risk score.

^a^ OR per SD increase; *P* values were estimated using logistic regression.

^b^ The NGRS was derived from body mass index, menopause status, waist-to-hip ratio, a previous diagnosis of benign breast disease, age at menarche, age at first live birth, family history of breast cancer, and an interaction term between body mass index and menopause status. Weights of these factors were derived from the training set including 416 individuals with breast cancer and 1558 control participants from the Shanghai Women’s Health Study.

^c^ Results for PRS111, the best PRS derived in the present study, are presented for comparison purposes. IRPM111 was the model including PRS111 and the NGRS.

^d^ Results for PRS263-meta, which was derived based on meta-analysis results of Asian and Breast Cancer Association Consortium–European data for 330 single-nucleotide variants initially reported in populations with European ancestry (Zhang et al^11^), are presented for comparison purposes. IRPM263-meta was the model including PRS263-meta and the NGRS.

### Absolute Risk of Developing Breast Cancer According to PRS Percentiles

**Figure 2.**
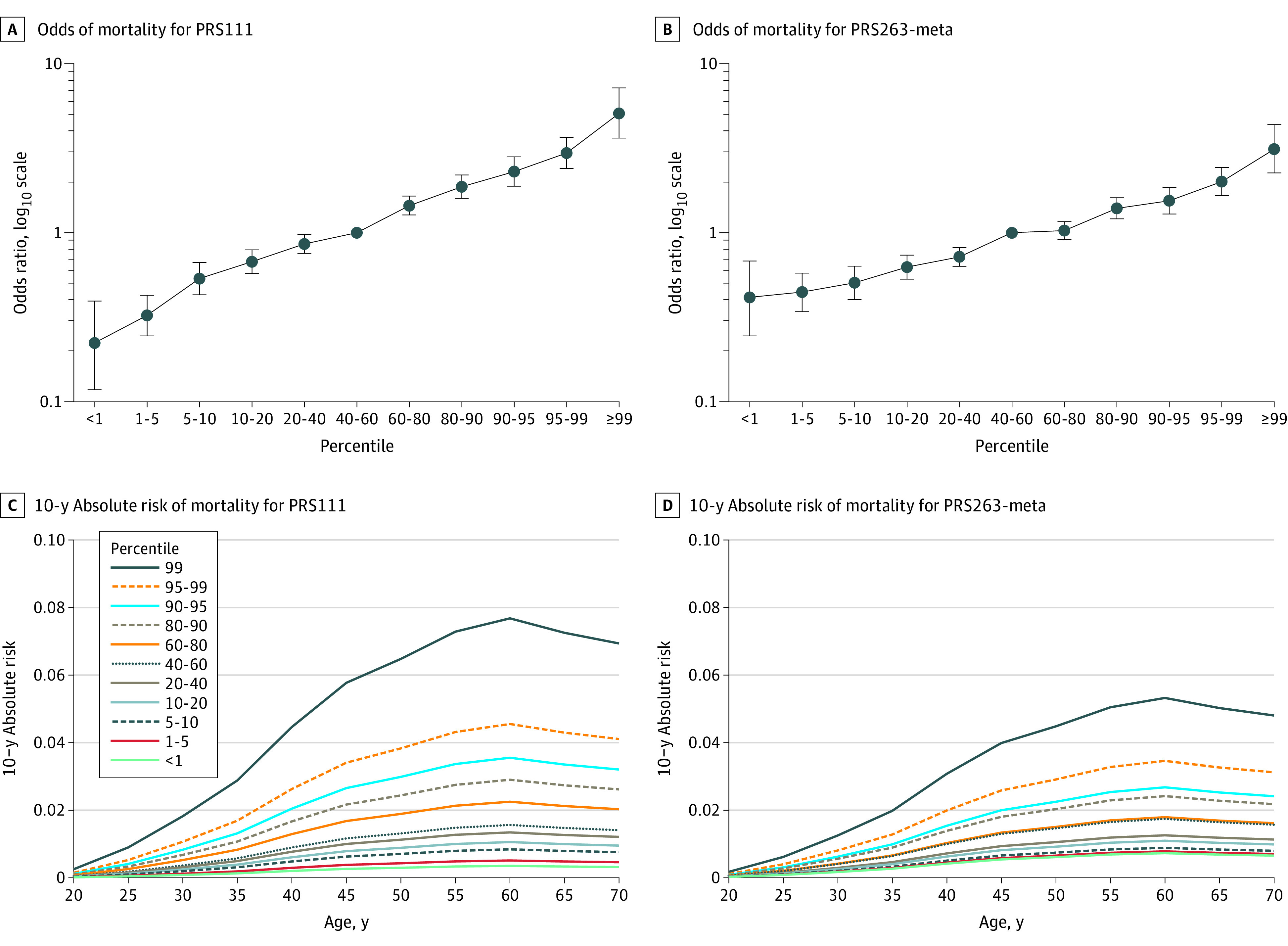
Ten-Year Absolute Risk of Developing Breast Cancer Estimated Using Data From 10 207 Chinese Women A and B, Odds ratios of breast cancer for percentiles of scores on the polygenic risk score using 111 single-nucleotide variants (PRS111) and the polygenic risk score based on a meta-analysis of European and Asian data (PRS263-meta) compared with the average risk group (ie, 40th-60th percentile). C and D, Ten-year absolute risk of breast cancer by percentiles of PRS111 and PRS263-meta score for women in different age categories.

Among the 10 207 Chinese women from the ABCC data sets, a dose-response association of breast cancer risk with percentiles of PRS111 and PRS263-meta was observed (Figure 2A and B). Compared with the average risk group, women in the top 5% of PRS111 and PRS263-meta were at a 3.39-fold (95% CI, 2.80-4.10) and 2.23-fold (95% CI, 1.87-2.65) increased risk of breast cancer, respectively; while those at the bottom 5% were at 0.30-fold (95% CI, 0.24-0.39) and 0.44-fold (95% CI, 0.35-0.56) decreased risk of breast cancer, respectively (eTable 7 in the Supplement). The 10-year absolute risks were estimated by PRS111 and PRS263-meta percentiles and age groups. For women aged 60 years, the ranges of 10-year absolute risks estimated by PRS111 and PRS263-meta were 0.35% to 7.68% and 0.73% to 5.32%, respectively (Figure 2C and D)

## Discussion

Leveraging large GWAS data sets of women with Asian ancestry, we developed PRSs for breast cancer risk using 3 approaches and validated their prediction performance in an independent prospective test cohort. PRS111, derived using the fine-mapping approach, was the best-performing PRS in this study (AUC, 0.639). The prediction model incorporating PRS111 and 7 nongenetic risk factors achieved a further improved prediction accuracy (AUC, 0.648).

A recent study compared the predictive ability of 5 Asian-specific PRSs with that of the 313-SNV European PRS in Asian women.^21^ The 313-SNV PRS was significantly more predictive (AUC, 0.617) than any of the 5 Asian-specific PRSs (best AUC, 0.586).^21^ However, because most of the breast cancer risk variants were identified in GWAS conducted among women with European ancestry, the Asian-specific PRSs were derived using a limited number of SNVs (from 5 to 51) in that study. In the present study, the most predictive PRS based on these 313 SNVs, PRS263-meta, underperformed the PRS111, which was derived entirely using Asian data. The prediction ability of PRS111 in Asian women (AUC, 0.639) is almost equivalent with that of the 313-SNV PRS in women with European ancestry (AUC, 0.642).^12^ The prediction ability of our PRS111 is also comparable with that derived using genomewide data in a recent study conducted among Asian women (AUC, 0.635).^34^

Most studies of prediction models incorporating PRS and nongenetic risk factors were carried out among European descendants.^16,24,35,36,37,38^ Generally, including nongenetic risk factors could lead to improved prediction accuracy, although the magnitude of improvement is relatively small. In a recent analysis using data from a prospective cohort of Dutch women, the 313-SNV European PRS was found to have an AUC of 0.636.^37^ Incorporating this PRS with 9 nongenetic risk factors improved the AUC to 0.653,^37^ similar to the level achieved in our study. In 2010, we built an Asian-specific prediction model incorporating a 12-SNV PRS and 5 nongenetic risk factors, which showed an AUC of 0.629 among Chinese women.^17^ In the present study, IRPM111, the prediction model including PRS111 and the NGRS, outperformed both PRS111 and the NGRS in predicting breast cancer risk.

The strengths of this study include the use of large GWAS data sets as the training set to improve the accuracy of estimating weights of breast cancer–associated SNVs for PRS construction. We performed fine-mapping analyses to identify additional breast cancer risk SNVs specifically for Asian women. Because most of the breast cancer–associated SNVs were identified in populations with European ancestry and there are differences in genetic architectures between Asian and European populations, we believe that this approach is necessary to construct a PRS that is more appropriate for Asian women. In addition, we demonstrated the ability of PRS-CSx in developing more predictive PRSs than other genomewide prediction algorithms. Finally, the availability of both genetic and nongenetic risk factors data made it possible to establish and validate prediction models incorporating PRSs and nongenetic risk factors.

### Limitations

This study has limitations. The sample size of our prospective test set was relatively small, which led to relatively wide 95% CIs for ORs and AUCs. Although the PRSs used for relative risk estimation were externally validated, there might still be some potential for overfitting in risk estimation. We found that all PRSs had better prediction performance in our prospective test set than in our case-control validation set. Reasons for this observation are unclear. Case and control participants were from 2 different studies in the case-control validation set, which could reduce the comparability between the case and control groups. However, the patterns of associations uncovered in both validation tests were similar. Additionally, including additional nongenetic factors, such as mammographic density, could improve model performance; however, such data were not available in the present study.

## Conclusions

Using data from the largest GWAS conducted in Asian women, we demonstrated that PRSs derived using breast cancer–associated risk SNVs show similar performance in predicting breast cancer risk in Asian and European descendants. Including known nongenetic risk factors in the models could further improve the accuracy of risk prediction. Our study provides strong support for the utility of risk prediction models in developing personalized screening and prevention strategies.

## Notes

Supplement. **eMethods.** Detailed Methods **eFigure.** Overall Workflow of the Fine-Mapping Strategy to Select SNVs for PRS Development **eTable 1.** Genotyping Platforms of ABCC Data Sets Contributing to the Current Study **eTable 2.** Characteristics of Participants From the Prospective Cohort Study SWHS Included in the Present Study **eTable 3.** Associations of PRSs With Breast Cancer Risk in the Validation Set, the Prospective Test Set, and the Combined Validation and Prospective Test Data Sets **eTable 4.** Associations With Breast Cancer Risk for the 263 SNVs in the ABCC Training Set, BCAC-European Data, and Meta-analyses **eTable 5.** Associations of the 111 SNVs in PRS111 With Breast Cancer Risk in the ABCC Training Set and BCAC-European Data **eTable 6.** Associations of PRS111 With Breast Cancer Risk Stratified by Age Categories in the Prospective Test Set **eTable 7.** Association With Breast Cancer Risk for PRS111 and PRS263-meta in 10 207 Chinese Women
eReferences.
