# Supplementary material for: Incorporating Polygenic Risk Scores and Nongenetic Risk Factors for Breast Cancer Risk Prediction Among Asian Women
Source: JAMA Netw Open. 2022 Mar 21;5(3):e2149030. doi: 10.1001/jamanetworkopen.2021.49030 (PMC8938714; doi:10.1001/jamanetworkopen.2021.49030)
Supplement: Supplement. — eMethods. Detailed Methods eFigure. Overall Workflow of the Fine-Mapping Strategy to Select SNVs for PRS Development eTable 1. Genotyping Platforms of ABCC Data Sets Contributing to the Current Study eTable 2. Characteristics of Participants From the Prospective Cohort Study SWHS Included in the Present Study eTable 3. Associations of PRSs With Breast Cancer Risk in the Validation Set, the Prospective Test Set, and the Combined Validation and Prospective Test Data Sets eTable 4. Associations With Breast Cancer Risk for the 263 SNVs in the ABCC Training Set, BCAC-European Data, and Meta-analyses eTable 5. Associations of the 111 SNVs in PRS111 With Breast Cancer Risk in the ABCC Training Set and BCAC-European Data eTable 6. Associations of PRS111 With Breast Cancer Risk Stratified by Age Categories in the Prospective Test Set eTable 7. Association With Breast Cancer Risk for PRS111 and PRS263-meta in 10 207 Chinese Women eReferences. [file jamanetwopen-e2149030-s001.pdf]

Supplemental Online Content

Yang Y, Tao R, Shu X, et al. Incorporating polygenic risk scores and nongenetic risk factors for breast cancer risk prediction among Asian women. *JAMA Netw Open.* 2022;5(3):e2149030. doi:10.1001/jamanetworkopen.2021.49030

eMethods. Detailed Methods

eFigure. Overall Workflow of the Fine-Mapping Strategy to Select SNVs for PRS Development

eTable 1. Genotyping Platforms of ABCC Data Sets Contributing to the Current Study

eTable 2. Characteristics of Participants From the Prospective Cohort Study SWHS Included in the Present Study

eTable 3. Associations of PRSs With Breast Cancer Risk in the Validation Set, the Prospective Test Set, and the Combined Validation and Prospective Test Data Sets

eTable 4. Associations With Breast Cancer Risk for the 263 SNVs in the ABCC Training Set, BCAC-European Data, and Meta-analyses

eTable 5. Associations of the 111 SNVs in PRS111 With Breast Cancer Risk in the ABCC Training Set and BCAC-European Data

eTable 6. Associations of PRS111 With Breast Cancer Risk Stratified by Age Categories in the Prospective Test Set

eTable 7. Association With Breast Cancer Risk for PRS111 and PRS263-meta in 10 207 Chinese Women

eReferences.

This supplemental material has been provided by the authors to give readers additional information about their work.

eMethods. Detailed Methods

Study participants

As shown in **Table 1**, the PRS development datasets included GWAS data of 20,076 breast cancer cases and 105,714 controls of Asian ancestry from the Asia Breast Cancer Consortium (ABCC), which was divided into a training set and a validation set. Detailed information on the ABCC is described elsewhere [1]. Study participants included in our training set were from six sources: the Shanghai Breast Cancer Genetics Study (SBCGS), including 11,731 Chinese women (5,384 cases and 6,347 controls) who were participants of four sub-studies; the Hwasun Cancer Epidemiology Study-Breast (HCES-Br), including 547 Korean women (274 cases and 273 controls); the Korea Precision Oncology Program (KPOP)-Breast Cancer, including 1,884 Korean women (963 cases and 921 controls); The Biobank Japan Project 2 (BBJ2), including 95,283 Japanese women (5,552 cases and 89,731 controls); the Seoul Breast Cancer Study (SeBCS), including 4,298 Korean women (2,246 cases and 2,052 controls); and eight other studies within the Breast Cancer Association Consortium (BCAC)-Asian data including 9,298 Asian-ancestry women (4,231 cases and 5,067 controls). PRSs developed using the training set were evaluated in an independent validation set from SBCGS, including 2,749 Chinese women, comprised of 1,426 cases from the Shanghai Breast Cancer Survival Study (SBCSS) [2], and 1,323 controls from the Shanghai Women's Health Study (SWHS) [3].

For each PRS development strategy, the most predictive PRSs in our validation set were further validated in an independent prospective test set comprising 368 cases and 736 individually matched controls (age ±five years old). All of these subjects were participants from the SWHS and did not have any diagnosis of any cancers at the time of enrollment [1,3]. In brief, during 1997 and 2000, the SWHS recruited approximately 75,000 adult women from urban Shanghai [3]. Incident cancer cases were identified via annual record linkage to the Shanghai Cancer Registry and in-person follow-up surveys, and confirmed by reviewing medical records [3]. All studies involved in the current analyses have been approved by their respective Institutional Review Boards and written informed consent has been obtained from all participants.

Genotyping, imputation, quality control, and GWAS

© 2022 Yang Y et al. *JAMA Network Open.*

Detailed descriptions of genotyping, quality control (QC), and imputation procedures are described in our recent publication [1]. Genotyping was conducted using several platforms (**eTable 1**) and genotyping data imputation was performed separately by study, with the 1000 Genomes Project Phase 3 (all populations) data as the reference panel. In our training set, GWAS was conducted within each study/sub-study using PLINK (v2.00a3LM) [4], adjusting for age, top five genetic principal components (PCs), and study (only for iCOGs and OncoArray datasets). Association results were combined via fixed-effects meta-analyses implemented in METAL (release 2018-08-28) [5].

In the present study, further QC steps were applied to data in our training set. First, except for the Exome BeadChip dataset that includes only ~50,000 SNPs, SNPs presented in less than half of the remaining eight datasets were excluded (**eTable 1**). Then, SNPs with an imputation quality of $R^2>0.80$ in MEGA datasets and $R^2>0.30$ in all the other datasets were retained. The reasons for imposing a more stringent threshold for MEGA datasets are twofold: (1) the MEGA array contains approximately 2.10 million variants (before imputation) with an excellent genomic coverage of common variants across multi-ethnic populations; (2) data in our validation and prospective test sets were genotyped using MEGA array. For PRS construction using GWAS data from both Asian- and European-ancestry populations, we included only SNPs with a minor allele frequency (MAF) of >0.01 in both East Asian and European subjects in the 1000 Genome Project Phase 3 data. In the end, a total of 5,947,015 SNPs were included in downstream analyses.

PRS development

In the present study, PRSs were calculated using the formula: $PRS = \sum_{k=0}^{n} \beta_k SNP_k$, in which SNP_k and β_k represent the allelic dosage and corresponding weight of $SNP\ k$, and n is the number of SNPs used.

Reported European PRS

For breast cancer, the best PRS to date was the one developed using 313 SNPs and their weights on breast cancer among European-ancestry women [6]. Most recently, this PRS was updated by adding 17 novel breast cancer susceptibility SNPs identified by GWAS among European-ancestry women [7]. Of these 330 SNPs, 263 could be found in our validation and prospective test sets, and three PRSs ($PRS_{263\text{-}ASN}$, $PRS_{263\text{-}EUR}$ and $PRS_{263\text{-}META}$) were

© 2022 Yang Y et al. *JAMA Network Open.*

derived using weights of these SNPs from our training set, BCAC-European data [7], and meta-analyses of these two datasets, respectively.

PRSs based on SNPs selected by fine-mapping of GWAS loci

The overall workflow of this strategy is presented in e**Figure 1**. A total of 245 susceptibility loci for breast cancer have previously been identified by GWAS, including 12 identified initially in GWAS among Asian-ancestry women only,[8-14] and 28 in our recent meta-analyses conducted among Asian- and European-ancestry women [15]. Of the 245 index SNPs in these loci, seven have a linkage disequilibrium (LD) with at least one of the remaining SNPs in either East Asians or Europeans (R^2>0.10); hence these seven variants were excluded. For each of the remaining 238 loci, fine-mapping analysis was performed using summary statistics of our training set to identify SNPs that were independently associated with breast cancer risk via the stepwise regression strategy implemented in GCTA-COJO (version 1.93.2.).[16] Genetic data of 504 subjects of East Asian ancestry included in the 1000 Genome Project Phase 3 were used as the reference for LD estimation. Within each locus, a COJO-*P* threshold of 10^{-5} was used to identify independent risk SNPs, weights of which on breast cancer risk were re-estimated via a joint analysis of all selected SNPs. Some loci were ineligible for fine-mapping because no SNPs within them had an association with breast cancer risk at $P<10^{-5}$ in our training set. Based on fine-mapping results, three PRSs were derived using (1) SNPs selected and weights re-estimated by fine-mapping; (2) SNPs selected by fine-mapping and showing a consistent association directions with $P<0.05$ in BCAC-European data [7], with weights re-estimated by fine-mapping; (3) SNPs and weights in (2), plus index SNPs from loci that were ineligible for fine-mapping and showed $P<0.05$ in our training set, with weights from our training set (**eFigure 1**). We repeated the fine-mapping analyses using COJO-*P* thresholds of 10^{-3} and 10^{-4} to identify independent risk variants, and used them to construct three PRSs for each threshold following the same steps described above.

PRSs based on genome-wide risk prediction algorithms

LDpred (version 1.0.11), LDpred2 (version 1.4.4), and PRS-CSx (July 29, 2021 release) were used to derive PRSs using genome-wide SNPs. The detailed description of these three algorithms can be obtained elsewhere [17-19]. In the present study, summary statistics of associations between 5,947,015 SNPs and breast cancer were used as the input to LDpred. Genetic data of 19,257 healthy women of East Asian ancestry were used as the reference panel for pair-

© 2022 Yang Y et al. *JAMA Network Open.*

wise LD estimation [1]. Of the 5,947,015 SNPs, indels, ambiguous SNPs, and SNPs with MAF<0.01 were further excluded by LDpred, and weights for the remaining 4,487,284 SNPs were re-evaluated with default settings. LDpred2, a strengthened version of LDpred released recently [19], recommends using SNPs included in HapMap3 [20] data since these SNPs have sufficient coverage of the whole genome [21]. Of the 5,948,258 SNPs included in our training set, 855,680 presents in Hapmap3 data, weights of which on breast cancer risk were re-estimated using LDpred2 with default settings. Distinct from LDpred and LDpred2, PRS-CSx reevaluates the weights of genome-wide SNPs through placing a continuous shrinkage prior on them, and is capable of improve cross-population polygenic prediction through integrating summary level GWAS data and external LD reference panels from multiple populations [22]. Usage of Hapmap3 SNPs is the default setting of PRS-CSx as well, thus the input for PRS-CSx was the same as that for LDpred2, including 855,680 SNPs. Five global shrinkage parameters, 1, 0.01, 1×10^{-4}, 1×10^{-6}, and 'auto' (automatically learning from the input data), were respectively used to reevaluate weights of the 855,680 SNPs on breast cancer risk.

Incorporation of PRSs and nongenetic risk factors

Established nongenetic breast cancer risk factors included body mass index (BMI), menopause status, waist-to-hip ratio (WHR), a previous diagnosis of benign breast disease, age at menarche, age at first live birth, and family history of breast cancer. An interaction term between BMI and menopause status was also included [23]. Data of 1,974 women (416 cases and 1558 controls) from the SWHS but independent from those in the prospective test set were used to estimate the weights of these seven nongenetic factors and the interaction term on breast cancer risk. A logistic regression model was fitted with case/control status of breast cancer as the outcome and these eight factors as predictors. Weights estimated from this model were then used to construct a nongenetic risk score (NgRS) for each subject in our prospective test set using the following formula:

$$NgRS = \sum_{k=0}^{7} w_k F_k \ + \ w_i BMI * Menopause$$

In this formula, F_k and w_k are the value and corresponding weight of factor k, and w_i is the weight of the interaction term between BMI and menopause status.

Prediction performance evaluation

© 2022 Yang Y et al. *JAMA Network Open.*

PRSs derived from the training set were first evaluated for their associations with breast cancer risk and prediction performance in our case-control validation set. Then, the most predictive PRSs from each PRS development approach were further appraised in our prospective test set. Finally, for the PRS showing the highest prediction accuracy in our prospective test set, and the reported European PRS,[6] an integrated risk prediction model (IRPM) was built including each PRS and the NgRS as independent predictors to predict breast cancer ($Breast\ cancer \sim PRS +\ NgRS$). Logistic regression was used to evaluate ORs and 95% confidence intervals (CIs) per standard deviation (SD) increase in these risk scores. Prediction performance was measured by AUCs and 95% CIs using the R (version 3.6.0; R Foundation) function *pROC:roc*.[24]

Stratified analyses by ancestry subgroups

Since all participants in validation and prospective test sets are Chinese, analyses stratified by ancestry subgroups could only be performed using subjects from the whole ABCC datasets. We excluded datasets that used Exome BeadChip or iCOGs for genotyping because these two platforms had relatively low genomic coverage (**eTable 1**). In the remaining datasets, individual genetic data were available for 10,207 Chinese women (Affymetrix, MEGA, OncoArray datasets in SBCGS) and 2,431 Korean women (HCES-Br and KPOP) (**eTable 1**). We evaluated associations with breast cancer risk and prediction performance of two PRSs, the most predictive PRS in the prospective test set and the reported European PRS [7], among these Chinese and Korean women through logistic regression and ROC analyses, respectively.

Absolute risk of developing breast cancer according to PRS percentiles

We estimated the 10-year absolute risk of developing breast cancer using the most predictive PRS in our prospective test set and the reported European PRS.[7] Considering that the prospective test set has a limited sample size (*N*=1,104), which would lead to unstable OR estimates, subjects from the whole ABCC datasets, except for the Exome BeadChip and iCOGs datasets (**eTable 1**), were also included. The aforementioned 10,207 Chinese women, including 5,087 cases and 5,120 controls, from the SBCGS and BCAC-Asians (**eTable 1**). Logistic regression was used to estimate breast cancer ORs of different PRS percentile groups compared to the middle quintile (40%-60%) group. Then 10-year absolute risks were calculated utilizing these ORs and the incidence and mortality rates of breast cancer in Shanghai following the strategy described previously [23].

© 2022 Yang Y et al. *JAMA Network Open.*

eFigure. Overall Workflow of the Fine-Mapping Strategy to Select SNVs for PRS Development

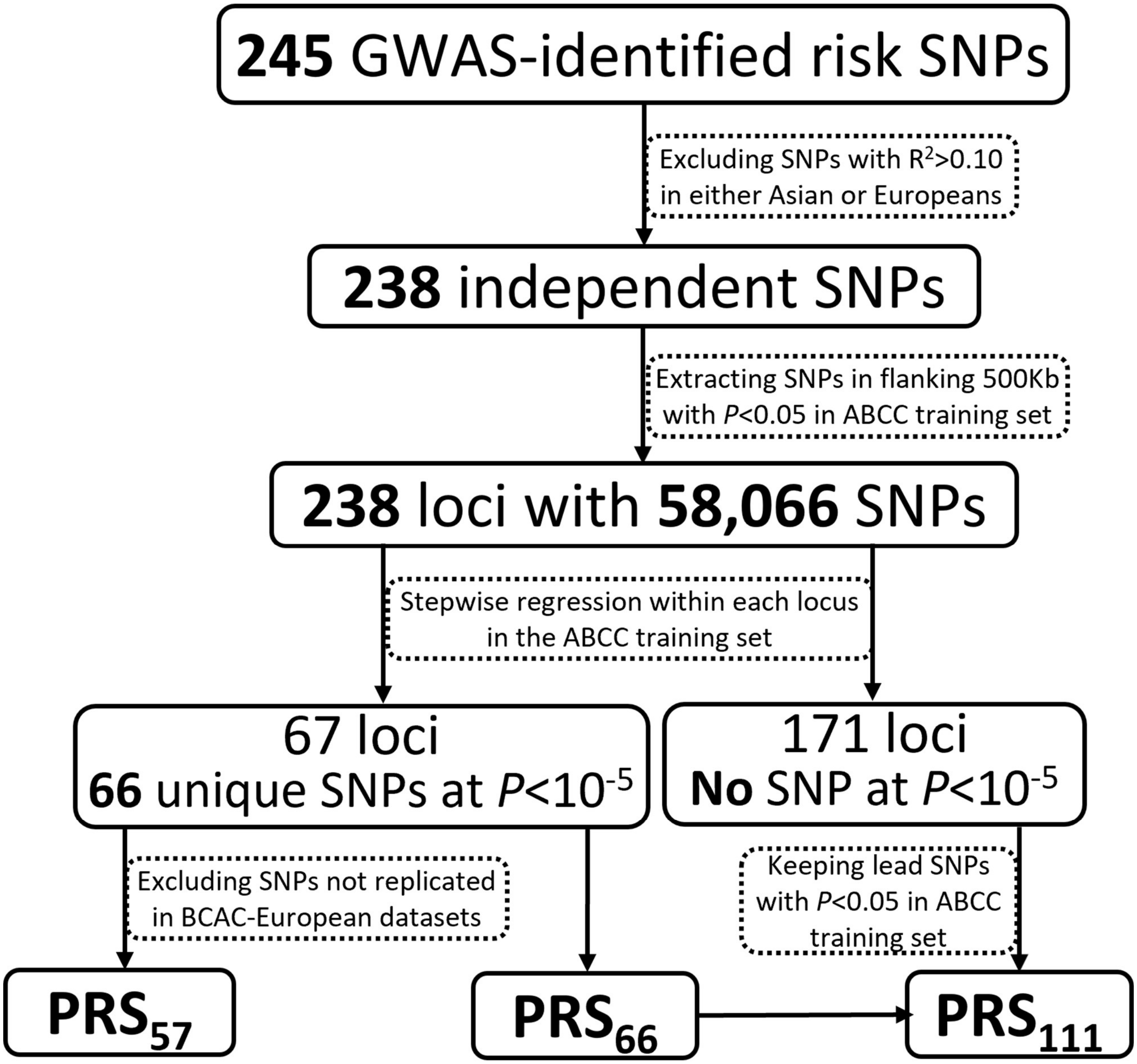

© 2022 Yang Y et al. *JAMA Network Open.*

eTable 1. Genotyping Platforms of ABCC Data Sets Contributing to the Current Study	
**Study**	**Sequencing platform**
**PRS development datasets**	
Training set	
SBCGS	
	Affymetrix GenomeWide Human SNP Array 6.0
	Illumina HumanExome-12v1_A Beadchip
	Illumina Infinium OncoArray-500K BeadChip
	Illumina iSelect Genotyping Array (iCOGs)
HCES-Br	Illumina Multi-Ethnic Genotyping Array
KPOP	Illumina Multi-Ethnic Genotyping Array
BBJ2	Illumina OmniExpress BeadChip
SeBCS	Affymetrix Genome-Wide Human SNP Array 6.0
BCAC-Asian	Illumina iSelect Genotyping Array (iCOGs)
Validation set	
SBCGS	Illumina Multi-Ethnic Genotyping Array
**Prospective test set**	
SBCGS	Illumina Multi-Ethnic Genotyping Array
Abbreviations: ABCC, Asia Breast Cancer Consortium; SBCGS, Shanghai Breast Cancer Genetic Study; HCES-Br, Hwasun Cancer Epidemiology Study-Breast; KPOP, Korea Precision Oncology Program; BBJ2, The Biobank Japan Project 2; SeBCS, Seoul Breast Cancer Study; BCAC, Breast Cancer Association Consortium	

© 2022 Yang Y et al. *JAMA Network Open.*

eTable 2. Characteristics of Participants From the Prospective Cohort Study SWHS Included in the Present Study

Characteristics	Categories	Training dataset for estimating weights of nongenetic factors - SWHS		Prospective test set - SWHS	
		Cases (*N*=416)	Controls (*N*=1,558)	Cases (*N*=368)	Controls (*N*=736)
Age at baseline interview					
	40-50	216 (51.9%)	815 (52.3%)	163 (44.3%)	365 (49.6%)
	50-60	91 (21.9%)	431 (27.7%)	117 (31.8%)	190 (25.8%)
	60-70	109 (26.2%)	312 (20.0%)	88 (23.9%)	181 (24.6%)
Age at menarche [a]		14.8 ± 1.8	14.9 ± 1.7	14.7 ± 1.6	14.9 ± 1.8 [b]
Age at first live birth [a]		27.2 ± 3.9	26.5 ± 3.9 [b]	26.8 ± 4.3	26.2 ± 3.8 [b]
Waist-to-hip ratio [a]		0.813 ± 0.053	0.816 ± 0.053	0.812 ± 0.053	0.810 ± 0.054
Body mass index [a]		24.2 ± 3.5	24.0 ± 3.3	24.5 ± 3.4	24.2 ± 3.5
Menopause status					
	Yes	184 (44.2%)	724 (46.5%)	193 (52.5%)	383 (52.0%)
	No	232 (55.8%)	834 (53.5%)	175 (47.5%)	353 (48.0%)
Family history of breast cancer					
	Yes	17 (4.1%)	29 (1.9%) [b]	19 (5.2%)	17 (2.3%) [b]
	No	399 (95.9%)	1,529 (98.1%)	349 (94.8%)	719 (97.7%)
History of benign breast disease					
	Yes	114 (27.4%)	281 (18.0%) [b]	63 (17.1%)	111 (15.1%)
	No	302 (72.6%)	1,277 (82.0%)	305 (82.9%)	625 (84.9%)

SWHS, Shanghai Women's Health Study.
[a] Mean ± standard deviation (SD) is presented.
[b] *P*<0.05 for case-control comparisons using t-tests for continuous variables and chi-squared tests for categorical variables.

© 2022 Yang Y et al. *JAMA Network Open.*

eTable 3. Associations of PRSs With Breast Cancer Risk in the Validation Set, the Prospective Test Set, and the Combined Validation and Prospective Test Data Sets												
**PRS development methods**	**Validation set (1,426 cases vs. 1,323 controls)**				**Prospective test set (368 cases vs. 736 controls)**				**Combined set (1,794 cases vs. 2,059 controls)**			
	**Mean (SD)**	**OR (95% CI) [a]**	**AUC (95% CI)**	***P* [a]**	**Mean (SD)**	**OR (95% CI) [a]**	**AUC (95% CI)**	***P* [a]**	**Mean (SD)**	**OR (95% CI) [a]**	**AUC (95% CI)**	***P* [a]**
**Published European PRS [b]**												
$PRS_{263\text{-}EUR}$	13.97(0.68)/ 13.74(0.64)	1.42 (1.31-1.53)	0.597 (0.575-0.618)	2.47 E-18	13.98(0.62)/ 13.68(0.64)	1.62 (1.42-1.85)	0.625 (0.590-0.659)	2.71 E-12	13.97(0.67)/ 13.72(0.64)	1.48 (1.38-1.58)	0.606 (0.588-0.623)	1.91 E-30
$PRS_{263\text{-}ASN}$	10.72(0.59)/ 10.51(0.57)	1.44 (1.33-1.56)	0.601 (0.580-0.622)	5.47 E-20	10.71(0.56)/ 10.46(0.55)	1.58 (1.38-1.80)	0.616 (0.582-0.651)	1.41 E-11	10.72(0.59)/ 10.49(0.56)	1.49 (1.40-1.60)	0.608 (0.590-0.626)	8.53 E-32
$PRS_{263\text{-}META}$	13.06(0.65)/ 12.83(0.61)	1.44 (1.33-1.55)	0.600 (0.579-0.621)	1.54 E-19	13.06(0.60)/ 12.77(0.60)	1.63 (1.43-1.87)	0.626 (0.592-0.661)	1.25 E-12	13.06(0.64)/ 12.81(0.61)	1.50 (1.40-1.60)	0.609 (0.591-0.626)	3.81 E-32
**Fine-mapping [c]**												
**COJO-*P*<10^{-3}**												
PRS_{219}	17.06(0.65)/ 16.88(0.64)	1.33 (1.23-1.44)	0.581 (0.560-0.603)	4.07 E-13	17.10(0.62)/ 16.83(0.62)	1.57 (1.38-1.80)	0.619 (0.584-0.654)	3.36 E-11	17.07(0.64)/ 16.86(0.63)	1.39 (1.31-1.49)	0.593 (0.575-0.611)	4.93 E-23
PRS_{120}	9.50(0.54)/9 .34(0.53)	1.36 (1.26-1.47)	0.587 (0.566-0.608)	4.78 E-15	9.52(0.51)/9 .30(0.50)	1.57 (1.38-1.80)	0.625 (0.590-0.661)	3.50 E-11	9.50(0.53)/9 .32(0.52)	1.42 (1.33-1.51)	0.599 (0.581-0.617)	4.84 E-25
PRS_{135}	10.16(0.55)/ 9.99(0.54)	1.38 (1.28-1.49)	0.592 (0.571-0.613)	3.30 E-16	10.18(0.52)/ 9.96(0.51)	1.54 (1.35-1.76)	0.619 (0.584-0.655)	1.55 E-10	10.17(0.54)/ 9.98(0.53)	1.42 (1.33-1.52)	0.600 (0.582-0.618)	9.46 E-26
**COJO-*P*<10^{-4}**												
PRS_{99}	8.87(0.52)/8 .72(0.52)	1.33 (1.23-1.44)	0.581 (0.560-0.602)	2.49 E-13	8.92(0.53)/8 .69(0.51)	1.60 (1.40-1.83)	0.622 (0.587-0.657)	4.77 E-12	8.88(0.52)/8 .71(0.52)	1.40 (1.31-1.50)	0.593 (0.575-0.611)	1.53 E-23
PRS_{73}	6.48(0.49)/6 .33(0.47)	1.37 (1.27-1.48)	0.588 (0.567-0.609)	1.85 E-15	6.51(0.47)/6 .30(0.45)	1.60 (1.40-1.83)	0.630 (0.595-0.665)	6.03 E-12	6.49(0.48)/6 .32(0.46)	1.43 (1.34-1.53)	0.600 (0.582-0.618)	5.29 E-26
PRS_{112}	8.11(0.51)/7 .93(0.50)	1.42 (1.31-1.53)	0.597 (0.575-0.618)	1.38 E-18	8.13(0.49)/7 .91(0.48)	1.63 (1.42-1.87)	0.632 (0.597-0.667)	1.70 E-12	8.11(0.50)/7 .92(0.49)	1.47 (1.38-1.57)	0.607 (0.589-0.624)	4.97 E-30
**COJO-*P*<10^{-5}**												

© 2022 Yang Y et al. *JAMA Network Open.*

PRS_{66}	6.36(0.48)/6.21(0.46)	1.37 (1.27-1.48)	0.590 (0.569-0.611)	1.30 E-15	6.39(0.47)/6.17(0.45)	1.66 (1.45-1.90)	0.634 (0.600-0.669)	1.91 E-13	6.37(0.48)/6.20(0.46)	1.45 (1.35-1.55)	0.603 (0.585-0.621)	9.91 E-28
PRS_{57}	5.33(0.46)/5.19(0.44)	1.38 (1.28-1.49)	0.591 (0.570-0.612)	4.87 E-16	5.36(0.43)/5.15(0.43)	1.66 (1.45-1.91)	0.641 (0.606-0.675)	2.16 E-13	5.34(0.45)/5.17(0.43)	1.45 (1.36-1.55)	0.605 (0.587-0.623)	4.12 E-28
PRS_{111}	7.61(0.49)/7.43(0.47)	1.45 (1.34-1.57)	0.603 (0.582-0.624)	2.72 E-20	7.63(0.47)/7.40(0.47)	1.67 (1.46-1.92)	0.639 (0.604-0.674)	1.28 E-13	7.61(0.49)/7.42(0.47)	1.51 (1.41-1.61)	0.614 (0.596-0.631)	4.22 E-33
**Genome-wide Bayesian algorithms** [d]												
PRS_{LDpred} (4,487,284 SNPs)	31.45(0.10)/31.41(0.10)	1.44 (1.34-1.56)	0.600 (0.579-0.621)	4.96 E-20	31.45(0.10)/31.41(0.10)	1.52 (1.34-1.74)	0.616 (0.581-0.651)	4.08 E-10	31.45(0.10)/31.41(0.10)	1.47 (1.37-1.57)	0.604 (0.586-0.622)	2.90 E-29
$PRS_{LDpred2}$ (855,680 SNPs)	52.26(0.37)/52.15(0.34)	1.40 (1.29-1.51)	0.591 (0.570-0.612)	4.77 E-17	52.28(0.36)/52.13(0.36)	1.51 (1.33-1.72)	0.612 (0.577-0.648)	7.47 E-10	52.27(0.37)/52.14(0.35)	1.43 (1.34-1.52)	0.597 (0.579-0.615)	7.17 E-26
$PRS_{PRS\text{-}CSx}$ (855,680 SNPs)	55.05(0.19)/54.98(0.18)	1.51 (1.39-1.63)	0.613 (0.592-0.634)	3.03 E-24	55.06(0.18)/54.97(0.18)	1.70 (1.49-1.95)	0.642 (0.608-0.676)	1.37 E-14	55.05(0.19)/54.97(0.18)	1.55 (1.45-1.66)	0.620 (0.602-0.637)	2.08 E-37

PRS, polygenic risk score; OR, odds ratio; CI, confidence interval; AUC, aera under the receiver operating characteristic curve.

[a] OR and 95% CI per standard deviation (SD) increase in PRS and *P* values was estimated using logistic regression.

[b] The 330-SNP European PRS reported by Zhang et al. *Nat Genet*. 2020. Based on 263 of the 330 SNPs that were available in our validation and prospective test sets, three PRSs were developed using weights from our training set, BCAC-European data, and meta-analyses these two datasets, respectively.

[c] At each COJO-*P* threshold, three PRSs were developed, respectively using (1) SNPs selected and weights re-estimated by fine-mapping; (2) SNPs from (1) and showing consistent association directions in BCAC-European data with *P*<0.05, with weights from fine-mapping; (3) SNPs and weights in (2), adding index SNPs in loci that were ineligible for fine-mapping and showing *P*<0.05 in our training set, and weights from our training set.

[d] For each algorithm, only the most predictive PRS in the validation set is presented. Weights of SNPs from our training set were re-estimated by each algorithm

© 2022 Yang Y et al. *JAMA Network Open.*

eTable 4. Associations With Breast Cancer Risk for the 263 SNVs in the ABCC Training Set, BCAC-European Data, and Meta-analyses

RSID	Chr	Position (GRCh37)	Effect allele	Non-effect allele	ABCC training set			BCAC-European data			Meta-analyses	
					Effect allele frequency (%)	Effect size	*P*	Effect allele frequency (%)	Effect size [b]	*P* [b]	Effect size	*P*
rs707475	1	7,917,076	A	G	19.35	0.00	1.00	41.95	-0.03	1.55E-07	-0.03	1.01E-06
rs616488	1	10,566,215	G	A	31.75	-0.05	2.52E-04	32.60	-0.06	9.62E-21	-0.06	1.70E-23
rs2992756	1	18,807,339	C	T	85.71	-0.04	6.83E-03	48.81	-0.05	2.67E-17	-0.05	7.10E-19
rs4233486	1	41,380,440	T	C	64.88	0.01	0.49	62.82	0.04	3.81E-08	0.03	1.57E-07
rs17426269	1	88,156,923	A	G	0.10	0.10	0.41	12.43	0.04	3.87E-07	0.04	3.04E-07
rs2151842	1	88,428,199	A	C	12.40	-0.02	0.18	26.94	-0.04	4.88E-09	-0.04	2.80E-09
rs612683	1	100,880,328	T	A	51.98	-0.01	0.45	38.17	0.03	3.62E-07	0.02	2.43E-05
rs7513707	1	114,445,880	A	G	58.63	0.02	0.19	19.18	0.06	3.54E-12	0.04	4.94E-11
rs12406858	1	118,141,492	C	A	46.03	0.02	0.11	27.24	0.04	2.14E-07	0.03	1.10E-07
rs637868	1	120,257,110	C	T	89.78	-0.04	0.11	53.58	0.04	5.89E-09	0.03	2.10E-07
rs11249433	1	121,280,613	G	A	2.98	0.09	0.01	43.14	0.10	1.21E-57	0.10	6.15E-59
rs143384623	1	145,604,302	CT	C	16.57	-0.01	0.74	34.00	-0.04	3.16E-10	-0.04	1.28E-09
rs11205303	1	149,906,413	C	T	28.37	0.03	0.06	36.88	0.05	5.07E-16	0.05	2.55E-16
rs12091730	1	155,556,971	A	G	66.67	0.05	1.71E-03	22.86	0.05	1.83E-12	0.05	1.27E-14
rs11463354	1	172,328,767	TA	T	13.10	0.01	0.53	32.31	-0.03	4.30E-05	-0.02	1.84E-04
rs6686987	1	202,184,600	T	C	25.89	-0.03	0.06	42.64	-0.01	0.04	-0.01	9.45E-03
rs7514172	1	203,770,448	A	T	32.44	0.06	8.86E-06	24.45	0.05	1.78E-12	0.05	9.19E-17

© 2022 Yang Y et al. *JAMA Network Open.*

rs2785646	1	208,076,291	A	G	2.18	-0.01	0.84	36.18	-0.03	5.12E-08	-0.03	5.56E-08
rs11117758	1	217,220,574	A	G	3.87	-0.03	0.32	22.76	-0.04	4.28E-09	-0.04	2.98E-09
rs11118563	1	220,671,050	T	C	35.22	-0.01	0.67	22.86	0.03	6.13E-05	0.02	1.06E-03
rs72755295	1	242,034,263	G	A	0.00	0.45	0.27	3.78	0.12	1.38E-12	0.12	1.03E-12
rs6743383	2	19,315,675	A	T	59.03	-0.04	1.04E-03	59.84	-0.04	1.91E-13	-0.04	8.50E-16
rs6725517	2	25,129,473	G	A	42.16	-0.02	0.15	39.36	-0.04	1.06E-09	-0.03	1.29E-09
rs12472404	2	29,179,452	C	G	80.06	0.00	0.84	22.76	0.00	0.77	0.00	0.73
rs9712235	2	67,881,757	A	G	81.05	0.00	0.83	75.94	-0.04	4.78E-08	-0.03	4.46E-07
rs4602255	2	69,392,128	A	G	89.29	0.02	0.44	47.71	0.04	1.95E-09	0.03	2.01E-09
rs6756513	2	70,172,587	A	G	29.46	-0.05	7.37E-05	27.34	-0.04	1.03E-07	-0.04	5.30E-11
rs1036759	2	88,358,825	C	G	27.08	0.02	0.32	32.41	0.03	1.64E-04	0.02	1.20E-04
rs6746250	2	121,058,254	G	A	40.28	-0.02	0.22	70.78	-0.03	1.38E-07	-0.03	1.64E-07
rs17625845	2	121,089,731	C	T	7.04	0.01	0.61	22.07	-0.04	1.89E-07	-0.03	2.42E-06
rs10164550	2	121,159,205	A	G	12.90	-0.01	0.51	37.38	-0.05	9.87E-14	-0.04	3.57E-13
rs10179592	2	121,246,568	C	T	79.07	0.08	7.55E-08	89.76	0.10	7.23E-23	0.09	4.79E-29
rs17726078	2	172,974,566	G	C	18.55	-0.01	0.37	45.63	-0.04	1.51E-11	-0.04	3.45E-11
rs1550622	2	174,212,910	G	A	99.50	0.14	0.07	84.69	0.05	6.75E-10	0.05	2.46E-10
rs2356656	2	192,381,934	T	C	94.64	-0.03	0.17	85.39	0.02	0.05	0.01	0.17
rs10197246	2	202,204,741	C	T	70.63	-0.09	4.38E-13	70.97	-0.06	4.38E-17	-0.06	3.79E-27
rs4442975	2	217,920,769	T	G	89.88	-0.08	3.30E-05	49.11	-0.13	1.03E-109	-0.13	8.91E-112
rs11693806	2	218,292,158	G	C	38.19	-0.06	1.27E-06	71.47	-0.07	2.75E-27	-0.07	3.39E-32
rs3791977	2	218,714,845	A	G	27.88	-0.02	0.10	34.99	-0.03	4.76E-07	-0.03	1.41E-07

© 2022 Yang Y et al. *JAMA Network Open.*

rs6762558	3	4,742,251	G	A	6.55	0.05	0.02	35.19	0.05	3.73E-19	0.05	2.16E-20
rs1375631	3	16,778,867	G	A	2.08	0.05	0.32	49.80	0.03	6.79E-09	0.03	4.19E-09
rs552647	3	27,353,716	A	C	26.59	0.07	1.65E-07	53.28	0.10	1.86E-67	0.10	3.25E-72
rs62255657	3	27,388,664	G	C	13.29	0.07	9.79E-06	29.42	0.10	5.01E-48	0.09	6.14E-52
rs17838698	3	30,684,907	T	C	69.15	0.04	2.39E-03	28.93	0.05	4.40E-14	0.05	5.74E-16
rs56387622	3	46,888,198	C	T	14.68	-0.05	3.34E-03	10.04	-0.09	9.68E-20	-0.08	1.76E-20
rs371314787	3	49,709,912	CT	C	6.35	-0.02	0.55	30.91	-0.02	6.02E-04	-0.02	5.04E-04
rs2886671	3	59,373,745	T	C	67.76	-0.01	0.66	40.46	-0.04	4.25E-08	-0.03	2.56E-07
rs147250346	3	63,887,449	TTG	T	13.99	0.02	0.42	14.31	0.06	1.60E-12	0.06	7.90E-12
rs9825432	3	71,620,370	G	T	3.27	-0.02	0.61	67.79	-0.04	4.88E-09	-0.04	5.00E-09
rs639355	3	99,403,877	A	G	47.92	0.00	0.80	51.89	-0.03	1.12E-08	-0.03	1.66E-07
rs376397524	3	141,112,859	C	CTT	7.44	0.05	0.07	40.06	0.05	4.38E-16	0.05	8.54E-17
rs58058861	3	172,285,237	A	G	32.74	0.03	0.03	17.89	0.05	2.40E-10	0.04	2.95E-11
rs9882792	3	189,774,456	T	C	10.42	0.01	0.63	20.87	-0.03	4.48E-05	-0.03	2.03E-04
rs495367	4	1,986,972	G	A	28.87	-0.01	0.54	30.62	0.04	5.28E-07	0.03	1.91E-05
rs10012017	4	38,784,633	T	G	48.12	0.02	0.21	27.83	0.05	7.66E-13	0.04	8.14E-12
rs532161833	4	84,370,124	TA	TAA	69.35	-0.03	0.08	51.79	-0.04	2.50E-12	-0.04	7.52E-13
rs17014016	4	89,240,476	A	G	1.19	0.09	0.13	42.84	0.04	2.47E-09	0.04	1.19E-09
rs62331150	4	106,069,013	T	G	63.59	0.00	0.71	21.17	0.04	4.09E-10	0.03	2.37E-08
rs147399132	4	126,752,992	AAT	A	35.91	0.01	0.46	50.60	-0.03	5.23E-07	-0.03	8.14E-06
rs56039025	4	143,467,195	T	C	2.68	0.00	0.93	12.03	-0.04	1.93E-05	-0.04	2.88E-05
rs138786872	4	151,218,296	C	CATATTT	26.09	-0.01	0.52	62.13	0.03	1.98E-07	0.03	7.69E-06

© 2022 Yang Y et al. *JAMA Network Open.*

rs28436676	4	175,842,495	A	G	23.81	-0.07	1.19E-07	10.83	-0.10	5.54E-27	-0.09	1.43E-32
rs62334414	4	175,847,436	A	C	1.79	0.04	0.31	35.09	0.05	3.02E-15	0.05	1.90E-15
rs10069690	5	1,279,790	T	C	16.87	0.06	3.52E-03	27.63	0.06	1.76E-18	0.06	2.36E-20
rs3215401	5	1,296,255	AG	A	38.39	-0.03	0.02	28.83	-0.07	1.13E-23	-0.06	5.12E-24
rs4866496	5	2,777,029	A	G	76.79	0.03	0.02	43.04	0.03	4.22E-07	0.03	2.19E-08
rs17611291	5	16,231,194	C	G	15.77	0.02	0.32	56.06	-0.04	2.89E-12	-0.04	5.26E-10
rs4613718	5	44,649,944	T	C	55.26	0.11	3.90E-19	61.63	0.05	5.85E-16	0.06	6.41E-29
rs10941679	5	44,706,498	G	A	48.71	0.10	1.96E-17	23.26	0.13	3.82E-84	0.13	7.98E-99
rs17343002	5	44,853,593	C	G	6.15	-0.12	3.12E-07	31.11	-0.05	7.12E-14	-0.05	9.53E-18
rs199562199	5	52,679,539	CA	C	19.54	0.02	0.28	11.23	0.05	1.81E-07	0.05	2.54E-07
rs553874618	5	55,662,540	CT	C	36.81	-0.02	0.09	39.76	-0.03	3.61E-06	-0.03	8.94E-07
rs889310	5	55,965,167	T	C	60.81	0.04	7.96E-04	58.15	0.04	3.32E-10	0.04	1.11E-12
rs16886165	5	56,023,083	G	T	34.03	0.06	7.29E-07	15.01	0.17	3.28E-104	0.14	2.26E-97
rs76250845	5	56,042,972	T	C	11.11	0.18	1.33E-18	4.87	0.20	3.48E-55	0.20	6.80E-72
rs11949391	5	56,045,081	C	T	5.16	-0.06	0.07	14.02	-0.09	1.50E-27	-0.09	4.89E-28
rs113778879	5	58,241,712	T	C	65.28	0.00	0.94	57.36	-0.04	1.70E-10	-0.03	6.33E-09
rs138044103	5	67,424,121	CTG	C	78.97	0.03	0.07	48.01	0.02	1.16E-04	0.02	2.13E-05
rs3010266	5	71,965,007	A	G	11.41	-0.05	9.64E-03	23.16	-0.04	7.44E-07	-0.04	2.85E-08
rs157557	5	73,234,583	C	T	42.16	0.01	0.48	32.01	-0.03	5.16E-05	-0.02	1.07E-03
rs144028731	5	77,155,397	G	GT	17.06	-0.03	0.12	37.67	-0.03	5.21E-06	-0.03	1.49E-06
rs34525310	5	79,180,995	GA	G	38.69	-0.01	0.39	15.01	0.03	3.55E-04	0.02	0.01
rs146817970	5	81,512,947	T	TA	0.00	-0.23	0.17	23.16	-0.05	3.91E-14	-0.05	2.64E-14

© 2022 Yang Y et al. *JAMA Network Open.*

rs332529	5	90,789,470	A	G	48.51	-0.07	8.33E-08	14.91	-0.06	6.63E-14	-0.06	3.17E-20
rs17157372	5	104,300,273	T	G	5.95	0.00	0.88	18.49	-0.02	2.90E-03	-0.02	4.25E-03
rs335160	5	122,478,676	A	C	61.90	0.00	1.00	77.34	-0.03	3.22E-06	-0.02	4.64E-05
rs1428387	5	122,705,244	T	C	8.43	0.03	0.14	2.68	0.09	3.91E-07	0.06	1.56E-06
rs6860806	5	131,640,536	G	A	30.46	0.01	0.46	52.88	0.03	2.66E-08	0.03	7.62E-08
rs6596100	5	132,407,058	T	C	9.13	-0.01	0.56	23.16	-0.04	1.70E-08	-0.04	3.98E-08
rs1432679	5	158,244,083	T	C	38.89	-0.07	9.14E-08	55.27	-0.07	1.56E-30	-0.07	9.11E-37
rs10074269	5	169,591,460	C	T	44.35	0.02	0.11	34.10	0.04	1.09E-09	0.04	8.05E-10
rs6864691	5	173,358,154	A	G	29.17	-0.01	0.68	40.16	0.03	3.29E-06	0.02	4.87E-05
rs418053	6	13,713,366	C	G	38.89	-0.04	6.77E-04	58.35	-0.05	1.56E-15	-0.05	5.21E-18
rs543824204	6	20,537,845	C	CA	31.65	-0.02	0.11	46.92	-0.04	1.61E-09	-0.04	6.29E-10
rs9358466	6	21,923,810	C	T	28.87	-0.02	0.13	45.23	-0.04	4.04E-09	-0.03	2.18E-09
rs17215231	6	33,239,869	T	C	2.38	-0.01	0.74	7.46	-0.03	0.01	-0.03	0.01
rs111342015	6	43,227,141	A	G	0.40	0.13	0.06	6.56	-0.05	1.27E-07	-0.05	7.59E-07
rs574103382	6	82,263,549	A	AAT	42.26	0.02	0.20	42.84	0.05	1.73E-14	0.04	9.48E-14
rs73754909	6	87,803,819	C	T	25.50	0.00	0.77	29.03	0.03	5.93E-05	0.02	4.53E-04
rs55941023	6	130,341,728	CT	C	93.15	0.02	0.51	70.78	0.04	3.92E-11	0.04	6.28E-11
rs2121348	6	149,595,505	C	T	42.96	-0.08	2.64E-12	18.39	-0.04	3.66E-08	-0.05	4.51E-17
rs6913578	6	151,949,806	C	A	33.93	0.20	1.51E-55	29.03	0.09	6.28E-47	0.11	3.17E-88
rs60954078	6	151,955,914	G	A	31.25	0.20	4.33E-50	7.95	0.18	8.74E-56	0.19	8.57E-104
rs851984	6	152,023,191	A	G	9.82	0.11	1.23E-09	41.75	0.06	3.31E-21	0.06	9.60E-28
rs6904031	6	152,055,978	T	A	6.85	0.14	7.16E-11	5.96	0.14	4.38E-29	0.14	2.42E-38

© 2022 Yang Y et al. *JAMA Network Open.*

rs910416	6	152,432,902	T	C	57.34	0.04	1.04E-03	54.77	0.06	4.48E-27	0.06	1.21E-28
rs9364472	6	169,006,947	G	C	36.90	0.01	0.55	54.87	-0.02	1.54E-04	-0.02	1.23E-03
rs6940159	6	170,332,621	C	T	14.78	0.07	2.43E-05	59.44	0.03	1.10E-07	0.04	9.23E-11
rs7971	7	21,940,960	G	A	16.77	-0.01	0.72	35.69	-0.04	1.69E-08	-0.03	8.81E-08
rs289997	7	25,569,548	T	C	24.70	0.01	0.67	15.81	-0.04	5.54E-07	-0.03	2.17E-05
rs13244925	7	55,192,256	C	A	68.85	-0.03	0.04	53.38	-0.03	2.41E-06	-0.03	2.58E-07
rs17268829	7	94,113,799	C	T	28.47	0.05	5.55E-04	28.83	0.05	2.58E-13	0.05	6.12E-16
rs111963714	7	99,948,655	G	T	3.08	0.00	0.94	19.98	0.03	1.17E-05	0.03	1.84E-05
rs71559437	7	101,552,440	A	G	7.04	-0.05	0.13	11.03	-0.06	1.19E-09	-0.06	3.80E-10
rs7800548	7	102,481,842	C	T	57.64	0.00	0.71	31.81	0.03	1.44E-07	0.03	6.38E-06
rs12706954	7	130,656,911	T	C	24.31	-0.01	0.49	35.39	-0.04	1.44E-11	-0.04	7.99E-11
rs68056147	7	130,674,481	A	G	26.39	0.05	9.85E-04	29.72	0.05	2.97E-15	0.05	1.24E-17
rs5887960	7	139,943,702	C	CT	54.17	0.05	8.48E-05	53.68	0.06	5.64E-18	0.05	2.38E-21
rs66823261	8	170,692	C	T	20.44	0.02	0.20	20.38	0.04	2.37E-06	0.03	1.41E-06
rs1028016	8	23,447,496	G	A	84.52	0.02	0.32	67.30	-0.03	1.84E-05	-0.02	1.75E-04
rs310295	8	23,663,653	A	C	29.96	0.03	0.02	39.56	0.03	1.43E-06	0.03	9.69E-08
rs13256025	8	25,831,778	T	C	1.39	0.02	0.62	22.56	0.04	1.41E-08	0.04	1.53E-08
rs9693444	8	29,509,616	C	A	70.04	-0.06	2.20E-06	65.41	-0.06	6.82E-21	-0.06	8.31E-26
rs13365225	8	36,858,483	G	A	31.25	-0.06	2.65E-06	14.21	-0.08	1.03E-21	-0.07	2.50E-26
rs1511243	8	76,230,943	G	A	98.51	0.11	0.01	83.60	0.08	2.20E-22	0.08	1.37E-23
rs1533366	8	76,378,165	T	G	25.99	-0.01	0.30	35.39	-0.04	4.33E-12	-0.04	1.37E-11
rs12546444	8	106,358,620	T	A	11.31	-0.08	1.10E-03	9.54	-0.07	1.08E-11	-0.07	4.90E-14

© 2022 Yang Y et al. *JAMA Network Open.*

rs13277568	8	116,679,547	G	A	44.05	0.00	0.75	35.79	-0.04	2.23E-08	-0.03	1.34E-06
rs13267382	8	117,209,548	G	A	47.92	-0.03	0.01	65.71	-0.04	7.65E-12	-0.04	3.69E-13
rs62526620	8	120,862,186	G	A	2.58	0.11	6.29E-03	11.13	0.04	3.20E-05	0.04	3.47E-06
rs35542655	8	124,563,705	C	T	18.25	0.03	0.07	15.21	0.06	1.12E-11	0.05	6.85E-12
rs12541094	8	124,571,581	A	G	36.41	0.01	0.43	41.35	0.03	2.47E-08	0.03	7.97E-08
rs7842619	8	124,739,913	G	T	24.21	0.02	0.20	39.86	0.04	3.04E-12	0.04	4.12E-12
rs12550713	8	128,370,949	G	C	51.98	0.04	3.45E-04	43.24	0.10	1.46E-65	0.09	7.36E-64
rs10096351	8	128,372,172	G	A	77.38	0.07	6.84E-06	56.76	0.11	3.77E-69	0.10	3.00E-72
rs1016578	8	129,199,566	A	G	19.15	-0.03	0.08	18.79	0.06	1.21E-15	0.04	5.29E-10
rs7830152	8	143,669,254	G	A	82.94	0.00	0.95	33.10	-0.02	3.31E-03	-0.02	6.07E-03
rs539723051	9	21,964,882	C	CAAAA	22.02	0.02	0.23	30.82	0.06	5.58E-22	0.06	3.16E-21
rs17694493	9	22,041,998	G	C	1.39	0.09	0.05	13.02	0.05	1.34E-08	0.05	2.73E-09
rs4880038	9	36,928,288	C	T	74.70	0.00	0.82	53.68	0.02	8.67E-05	0.02	4.75E-04
rs665889	9	87,782,211	C	T	78.37	0.01	0.62	52.78	0.02	1.49E-03	0.02	1.87E-03
rs10120432	9	98,362,587	C	T	34.42	0.01	0.55	8.55	0.04	5.16E-05	0.03	3.94E-04
rs4742903	9	106,856,793	C	G	20.34	0.03	0.08	57.65	0.03	2.58E-08	0.03	5.87E-09
rs60037937	9	110,303,808	T	TAA	43.25	0.03	8.98E-03	22.96	0.08	5.24E-26	0.07	2.42E-25
rs10816625	9	110,837,073	G	A	38.19	0.08	2.15E-10	9.34	0.11	4.20E-20	0.10	3.96E-28
rs13294895	9	110,837,176	T	C	2.08	0.04	0.44	18.89	0.06	5.00E-16	0.06	4.06E-16
rs630965	9	110,885,479	T	C	91.57	0.05	0.01	63.12	0.10	1.95E-59	0.10	8.55E-60
rs1895062	9	119,313,486	G	A	30.36	0.00	0.83	42.45	-0.04	2.51E-12	-0.04	1.33E-10
rs3861871	9	129,424,719	G	A	57.94	-0.06	1.40E-06	46.42	-0.03	4.75E-08	-0.04	2.16E-12

© 2022 Yang Y et al. *JAMA Network Open.*

rs550057	9	136,146,597	T	C	19.05	0.01	0.43	28.23	0.03	3.81E-07	0.03	9.55E-07
rs55910451	10	5,794,652	G	A	15.97	0.02	0.26	20.08	0.04	6.93E-07	0.03	6.66E-07
rs10796139	10	13,892,298	A	G	61.51	0.01	0.64	44.93	0.03	2.22E-05	0.02	5.46E-05
rs7072776	10	22,032,942	G	A	94.54	0.01	0.79	71.37	-0.06	2.51E-21	-0.06	2.47E-20
rs10764337	10	22,861,490	C	A	92.86	-0.07	0.05	94.33	0.07	8.35E-09	0.06	2.37E-06
rs10995201	10	64,299,890	G	A	2.18	-0.06	0.11	15.21	-0.13	1.60E-49	-0.12	1.71E-49
rs6479868	10	64,819,996	T	G	16.77	0.03	0.04	19.38	0.03	1.44E-04	0.03	1.63E-05
rs111833376	10	71,335,574	T	C	9.72	0.04	0.09	28.23	-0.02	1.78E-03	-0.02	0.01
rs719338	10	80,851,257	T	G	66.47	-0.06	4.28E-06	57.95	-0.08	2.81E-37	-0.07	1.86E-41
rs4980029	10	80,886,726	G	A	46.43	0.06	1.85E-05	14.81	0.08	4.70E-22	0.07	1.12E-25
rs140936696	10	95,292,187	C	CAA	62.80	-0.01	0.32	82.50	-0.04	8.37E-07	-0.03	1.84E-06
rs10885405	10	114,777,670	T	C	2.98	0.07	0.02	49.20	0.05	3.30E-14	0.05	3.53E-15
rs12250948	10	115,128,491	C	T	24.70	-0.05	4.18E-05	78.33	-0.05	7.78E-14	-0.05	1.55E-17
rs9421410	10	123,095,209	A	G	40.48	-0.04	1.39E-03	33.30	-0.05	5.45E-13	-0.05	3.31E-15
rs45631580	10	123,340,107	G	A	11.31	0.04	0.04	7.16	-0.13	5.35E-26	-0.09	1.75E-15
rs35054928	10	123,340,431	G	GC	55.06	-0.18	3.94E-49	54.97	-0.25	0.00E+00	-0.23	3.87e-399
rs6597981	11	803,017	G	A	27.58	0.03	0.02	49.80	0.05	4.65E-14	0.04	5.43E-15
rs4980386	11	1,895,708	A	C	71.63	-0.08	5.13E-08	39.96	-0.08	4.89E-35	-0.08	1.62E-41
rs10832963	11	18,664,241	G	T	48.71	0.00	0.70	71.97	0.03	8.89E-06	0.03	5.27E-05
rs4472923	11	42,844,441	T	C	22.82	0.02	0.17	34.79	-0.01	0.07	-0.01	0.24
rs10838267	11	44,368,892	A	G	29.27	0.04	1.58E-03	51.69	0.03	4.51E-08	0.03	3.36E-10
rs77047825	11	46,318,032	G	C	0.00	-0.17	0.58	7.55	-0.04	8.34E-04	-0.04	7.72E-04

© 2022 Yang Y et al. *JAMA Network Open.*

rs12287832	11	65,553,492	A	C	11.11	0.03	0.10	18.49	0.05	1.45E-12	0.05	6.21E-13
rs10896047	11	65,572,431	A	G	22.72	-0.03	0.06	47.81	-0.04	2.89E-13	-0.04	7.99E-14
rs35039974	11	69,328,130	T	A	23.81	-0.02	0.14	18.29	-0.07	1.15E-22	-0.06	6.96E-21
rs661204	11	69,330,983	A	G	0.99	0.25	1.77E-03	12.23	0.22	8.00E-137	0.22	6.55E-139
rs78540526	11	69,331,418	T	C	0.50	0.26	0.02	6.76	0.28	2.09E-147	0.28	1.22E-148
rs7125780	11	103,614,438	G	T	66.37	-0.02	0.10	65.11	0.02	0.01	0.01	0.11
rs199504893	11	108,267,402	CA	C	38.69	0.00	0.79	45.33	0.00	0.84	0.00	0.76
rs610437	11	111,696,440	C	T	77.98	-0.03	0.03	61.53	-0.03	2.22E-07	-0.03	2.07E-08
rs625145	11	116,727,936	T	A	17.66	-0.02	0.27	16.60	-0.03	2.29E-04	-0.03	1.58E-04
rs7924772	11	120,233,626	G	A	24.21	0.03	0.08	38.87	0.02	6.93E-04	0.02	1.38E-04
rs7121616	11	122,966,626	G	A	36.11	-0.02	0.18	28.53	-0.03	3.32E-05	-0.03	1.75E-05
rs7939702	11	129,243,417	G	T	96.63	-0.04	0.41	84.99	-0.05	1.87E-08	-0.05	1.35E-08
rs11822830	11	129,461,016	G	A	51.88	0.03	0.02	55.96	0.05	2.72E-14	0.04	4.37E-15
rs797736	12	293,626	G	A	41.96	0.01	0.59	36.48	0.03	4.96E-05	0.02	1.24E-04
rs12422552	12	14,413,931	C	G	26.98	0.07	6.95E-07	29.03	0.06	8.12E-18	0.06	3.91E-23
rs788458	12	28,149,568	T	C	17.76	-0.13	1.19E-16	10.64	-0.15	5.89E-54	-0.14	1.06E-68
rs7297051	12	28,174,817	T	C	22.32	-0.12	1.75E-16	23.26	-0.12	2.38E-67	-0.12	4.21E-82
rs1027113	12	29,140,260	A	G	75.89	0.05	7.30E-04	92.84	0.07	7.67E-12	0.06	7.59E-14
rs2277339	12	57,146,069	G	T	20.63	-0.05	1.97E-03	11.13	-0.04	1.29E-04	-0.04	9.96E-07
rs2870876	12	70,798,355	T	A	34.72	-0.01	0.33	17.69	0.03	1.06E-04	0.02	5.00E-03
rs111622698	12	83,064,195	GA	G	15.67	0.00	0.85	11.73	0.06	1.61E-07	0.04	6.94E-06
rs10862899	12	85,004,551	T	C	11.90	-0.02	0.25	49.70	0.03	1.19E-08	0.03	5.43E-07

© 2022 Yang Y et al. *JAMA Network Open.*

rs17356907	12	96,027,759	G	A	26.39	-0.05	1.08E-03	29.32	-0.09	8.11E-41	-0.08	1.67E-41
rs1061657	12	115,108,136	C	T	41.67	0.01	0.49	28.13	0.04	2.52E-10	0.04	4.11E-09
rs11067551	12	115,796,577	G	A	29.07	-0.05	1.43E-04	20.28	-0.04	9.39E-08	-0.04	6.86E-11
rs2454399	12	115,835,836	C	T	25.99	-0.10	5.53E-14	41.35	-0.08	1.13E-42	-0.09	9.01E-55
rs2464195	12	121,435,475	A	G	48.02	-0.03	6.08E-03	37.77	-0.02	1.88E-03	-0.02	5.48E-05
rs9315973	13	43,501,356	G	A	61.61	-0.01	0.46	80.72	0.04	1.39E-05	0.02	8.02E-04
rs12870942	13	73,806,982	C	T	23.41	0.05	4.22E-04	30.42	0.04	3.79E-11	0.04	7.60E-14
rs2181965	13	73,960,952	G	A	99.50	-0.02	0.84	77.04	0.04	6.50E-10	0.04	8.00E-10
rs34914085	14	37,128,564	A	C	31.65	-0.07	7.18E-07	21.57	-0.07	2.66E-22	-0.07	1.18E-27
rs2253012	14	37,228,504	T	C	10.32	0.05	0.01	43.34	0.04	5.04E-10	0.04	2.27E-11
rs2588809	14	68,660,428	C	T	97.22	-0.08	0.02	81.21	-0.06	1.36E-14	-0.06	1.23E-15
rs11624333	14	68,979,835	C	T	5.56	-0.01	0.71	23.66	-0.10	1.55E-43	-0.09	2.03E-40
rs11341843	14	91,751,788	T	TC	49.31	0.03	0.03	67.69	0.05	1.57E-13	0.04	4.99E-14
rs941764	14	91,841,069	G	A	13.69	0.03	0.10	35.29	0.05	4.11E-15	0.05	1.87E-15
rs4983544	14	105,213,978	G	T	65.97	0.02	0.17	44.63	0.04	2.04E-09	0.03	2.06E-09
rs4774565	15	50,694,306	G	A	51.59	0.01	0.56	33.80	-0.03	2.76E-06	-0.03	2.65E-05
rs8042593	15	66,630,569	A	G	81.15	0.00	0.92	61.53	-0.03	1.61E-05	-0.02	5.92E-05
rs35874463	15	67,457,698	G	A	0.00	0.31	0.37	5.27	0.07	2.59E-07	0.07	2.19E-07
rs8035987	15	75,750,383	C	T	37.90	-0.05	1.76E-04	28.93	-0.03	5.94E-07	-0.04	5.98E-10
rs2290202	15	91,512,267	T	G	51.29	-0.07	3.34E-09	14.02	-0.08	1.04E-17	-0.07	2.36E-25
rs144767203	15	100,905,819	C	A	22.92	-0.03	0.15	9.54	-0.05	4.22E-06	-0.04	2.64E-06
rs11076805	16	4,106,788	A	C	11.41	-0.03	0.26	25.75	-0.03	1.14E-04	-0.03	5.77E-05

© 2022 Yang Y et al. *JAMA Network Open.*

rs12709163	16	6,963,972	G	C	88.79	-0.04	0.09	79.03	0.01	0.14	0.01	0.43
rs34872983	16	10,706,580	A	G	23.81	-0.02	0.15	5.07	-0.07	1.85E-07	-0.05	6.32E-07
rs75753503	16	23,007,047	T	G	3.27	-0.01	0.82	2.49	0.07	8.57E-04	0.05	4.95E-03
rs35668161	16	52,538,825	A	C	18.75	0.20	1.64E-37	27.24	0.21	4.88E-212	0.21	1.55E-247
rs4784227	16	52,599,188	T	C	25.50	0.21	1.18E-51	25.45	0.21	5.65E-214	0.21	1.34E-263
rs55872725	16	53,809,123	T	C	16.57	-0.07	1.52E-05	43.24	-0.06	2.20E-22	-0.06	2.09E-26
rs6499648	16	53,861,139	T	C	67.66	-0.03	0.03	78.53	-0.04	2.48E-09	-0.04	3.34E-10
rs7184573	16	53,861,592	A	G	18.85	-0.01	0.37	39.66	-0.05	3.20E-14	-0.04	1.33E-13
rs28539243	16	54,682,064	A	G	58.04	0.03	8.55E-03	47.91	0.05	1.52E-14	0.04	7.63E-16
rs7500067	16	80,648,296	G	A	25.99	0.04	4.89E-03	24.65	0.08	1.68E-30	0.07	2.86E-30
rs9931038	16	85,145,977	C	T	11.61	0.00	0.94	46.12	-0.01	0.08	-0.01	0.09
rs12449271	16	87,086,492	C	T	20.14	-0.03	0.06	26.44	-0.04	9.24E-10	-0.04	1.96E-10
rs79461387	17	29,168,077	T	G	9.52	-0.06	0.06	27.63	-0.04	1.22E-09	-0.04	2.22E-10
rs545502941	17	43,212,339	CT	C	9.72	-0.02	0.34	22.76	0.03	7.28E-05	0.02	8.11E-04
rs559816018	17	44,283,858	A	G	0.10	-0.12	0.54	19.48	-0.05	1.68E-11	-0.05	1.47E-11
rs2787486	17	53,209,774	C	A	27.98	-0.08	4.02E-09	28.63	-0.07	1.46E-27	-0.07	4.99E-35
rs11652463	17	70,405,095	G	C	74.80	-0.01	0.30	31.01	-0.04	4.19E-08	-0.03	8.09E-08
rs745570	17	77,781,725	G	A	41.27	-0.04	4.48E-03	49.50	-0.04	1.56E-10	-0.04	2.58E-12
rs206435	18	10,354,649	C	A	49.70	0.00	0.85	50.00	0.00	0.99	0.00	0.93
rs16976596	18	11,696,613	T	C	2.98	-0.05	0.46	14.91	-0.03	3.34E-03	-0.03	2.59E-03
rs11665269	18	20,634,253	T	C	48.61	0.01	0.66	62.43	-0.04	2.35E-08	-0.03	2.19E-06
rs1111207	18	24,125,857	C	T	66.37	0.02	0.09	43.14	0.03	6.44E-08	0.03	2.00E-08
rs527616	18	24,337,424	G	C	74.21	0.04	3.69E-03	60.44	0.05	1.68E-16	0.05	3.41E-18

© 2022 Yang Y et al. *JAMA Network Open.*

rs35369219	18	24,518,050	A	AT	49.21	-0.06	1.87E-06	27.34	-0.06	1.67E-20	-0.06	1.75E-25
rs8092192	18	25,407,513	G	C	45.44	0.01	0.48	70.87	0.03	7.84E-06	0.02	2.05E-05
rs72931898	18	29,981,526	A	G	1.09	-0.07	0.31	4.08	-0.10	9.68E-13	-0.10	6.24E-13
rs9954058	18	42,411,803	C	G	13.49	-0.08	1.06E-05	7.26	-0.08	1.30E-12	-0.08	6.83E-17
rs9952980	18	42,888,797	C	T	30.95	-0.09	1.96E-12	34.00	-0.05	1.08E-14	-0.06	8.31E-24
rs56069439	19	17,393,925	A	C	0.20	-0.05	0.67	26.04	0.04	8.98E-09	0.04	1.10E-08
rs10164323	19	18,569,492	T	C	21.63	-0.05	2.54E-04	35.09	-0.07	2.08E-27	-0.07	5.53E-30
rs140702307	19	19,517,054	CGGGCG	C	40.67	0.04	9.18E-03	34.00	0.04	4.14E-10	0.04	1.32E-11
rs56681946	19	44,283,031	C	T	15.08	-0.01	0.58	34.19	0.06	2.34E-22	0.05	1.32E-19
rs4399645	19	46,166,073	C	T	40.18	-0.03	0.06	60.64	-0.04	3.37E-08	-0.03	6.26E-09
rs1172821	19	55,816,678	T	C	9.82	-0.02	0.35	35.59	-0.03	3.52E-06	-0.03	2.29E-06
rs16991615	20	5,948,227	A	G	0.00	0.13	0.75	6.66	0.08	7.48E-10	0.08	7.18E-10
rs6065254	20	39,248,265	A	G	39.48	-0.01	0.32	41.05	-0.03	1.99E-05	-0.02	1.99E-05
rs6030585	20	41,613,706	G	C	84.52	-0.02	0.18	80.42	0.02	0.04	0.01	0.16
rs13039563	20	52,296,849	A	G	32.04	0.04	4.88E-03	24.25	0.04	3.05E-09	0.04	5.48E-11
rs2822999	21	16,364,756	G	T	15.18	0.00	1.00	16.90	0.06	2.86E-12	0.05	6.88E-10
rs2823130	21	16,566,350	G	A	12.90	0.07	1.05E-04	9.44	0.07	3.52E-10	0.07	1.66E-13
rs2403907	21	16,574,455	A	C	11.61	-0.04	0.03	29.22	-0.08	1.38E-33	-0.07	7.99E-34
rs4818836	21	47,762,932	A	G	0.10	0.24	7.59E-03	3.78	0.08	8.81E-07	0.09	1.02E-07
rs9798754	22	19,766,137	T	C	58.63	-0.04	2.53E-03	36.38	-0.03	6.59E-08	-0.04	6.64E-10
rs5997390	22	29,135,543	A	G	6.35	0.07	1.02E-03	7.95	0.07	2.42E-12	0.07	1.02E-14
rs536920426	22	38,583,315	AAAAGAAAG	AAAAG	23.51	-0.01	0.61	29.42	-0.05	4.32E-13	-0.05	3.33E-12

© 2022 Yang Y et al. *JAMA Network Open.*

rs5750715	22	39,343,916	A	T	47.12	0.06	4.29E-07	27.44	0.04	2.05E-10	0.05	1.16E-15
rs66987842	22	40,904,707	C	CT	23.91	0.06	2.83E-06	10.44	0.12	7.18E-36	0.10	3.30E-38
rs112855987	22	45,319,953	A	G	29.76	-0.03	0.04	41.75	-0.01	0.08	-0.01	0.02

[a] The 330-SNP European PRS for breast cancer reported by Zhang et al. *Nat Genet*. 2020. Of the 330 SNPs, 263 SNPs were available in our validation and prospective test sets. Based on these 263 SNPs, $PRS_{263\text{-}ASN}$, $PRS_{263\text{-}EUR}$ and $PRS_{263\text{-}META}$ were derived using weights from our training set, BCAC-European data, and meta-analyses of these two datasets respectively.
[b] BCAC-European data from Zhang et al. *Nat Genet*. 2020.

© 2022 Yang Y et al. *JAMA Network Open.*

eTable 5. Associations of the 111 SNVs in PRS111 With Breast Cancer Risk in the ABCC Training Set and BCAC-European Data

RSID	Chr	Position (GRCh37)	Effect allele	Non-effect allele	ABCC training set			BCAC-European data		
					Effect allele frequency (%)	Effect size	*P*	Effect allele frequency (%)	Effect size [c]	*P* [c]
**57 SNPs selected by fine-mapping at COJO-*P*<10^{-5} using our training set [a]**										
rs4846235	1	10,632,235	T	C	20.24	-0.080	2.13E-07	31.11	-0.06	6.46E-21
rs7529564	1	156,189,793	T	C	86.21	-0.072	7.54E-06	63.82	-0.03	1.18E-06
rs67087079	1	203,850,783	A	G	24.60	0.072	1.89E-07	11.43	0.05	1.01E-09
rs12127615	1	88,181,633	A	G	22.32	-0.068	7.36E-06	59.24	-0.01	0.03
rs4848601	2	121,243,011	C	G	79.07	0.086	3.23E-08	89.76	0.10	2.17E-22
rs10931936	2	202,143,928	T	C	30.36	0.103	6.24E-16	28.33	0.06	2.72E-17
rs57481445	2	218,296,374	A	G	41.77	-0.064	8.68E-08	72.56	-0.07	5.57E-26
rs34197427	3	27,532,310	A	G	14.19	0.096	4.70E-08	37.57	0.05	1.01E-14
rs6440015	3	141,336,351	T	G	29.56	0.061	2.12E-06	37.97	0.03	2.46E-07
rs73010941	3	150,474,477	T	C	56.15	0.103	4.19E-16	97.22	0.05	7.33E-03
rs2945330	4	48,606,526	A	C	62.80	0.066	1.51E-07	48.91	0.02	6.79E-04
rs9884717	4	175,833,091	A	G	76.59	0.081	1.04E-08	89.17	0.10	6.25E-27
rs4339357	5	44,670,741	T	C	45.24	-0.104	4.23E-18	59.15	-0.09	3.05E-53
rs112776581	5	56,054,333	T	TA	11.01	0.194	1.11E-20	4.87	0.20	5.02E-55
rs6860948	5	90,696,297	T	G	49.40	0.071	6.53E-09	85.79	0.06	1.35E-12
rs17715065	5	158,261,163	T	C	38.19	-0.068	4.37E-08	49.30	-0.07	4.93E-29
rs2444832	6	81,339,849	A	T	31.85	0.057	5.12E-06	38.97	0.02	2.26E-03
rs9444166	6	85,088,902	A	C	79.96	0.072	2.12E-06	68.39	0.03	9.02E-05
rs4897114	6	149,607,978	A	G	42.56	-0.084	1.41E-12	16.40	-0.04	1.71E-06
rs7763637	6	151,949,312	A	G	34.03	0.215	1.99E-62	29.82	0.09	1.30E-44
rs862346	6	152,016,369	A	T	87.70	-0.083	4.38E-07	55.27	-0.05	1.47E-14
rs79388591	6	152,355,649	G	GT	67.46	-0.096	1.52E-13	92.15	-0.04	3.22E-04
rs9397082	6	152,430,638	T	C	36.11	0.063	2.91E-07	27.63	0.05	3.69E-12

© 2022 Yang Y et al. *JAMA Network Open.*

rs2172905	6	170,334,502	T	C	78.77	-0.067	4.37E-06	28.83	-0.03	8.31E-06
rs17164125	7	91,417,796	T	C	61.61	0.062	5.99E-07	90.85	0.03	2.19E-03
rs13235624	7	139,943,267	T	C	46.43	0.057	4.47E-06	43.24	0.05	2.81E-17
rs4732987	8	29,494,941	T	C	51.69	0.062	4.65E-07	60.54	0.03	2.90E-07
rs146992477	8	36,842,055	T	TTCTTTCTTTC	68.95	0.069	1.64E-07	86.28	0.07	4.28E-18
rs34302508	8	102,654,384	CT	C	91.07	0.112	1.71E-07	96.82	0.04	0.02
rs2392780	8	128,388,025	A	G	73.81	0.073	1.84E-07	58.95	0.10	5.92E-63
rs1333035	9	22,044,059	A	G	81.94	-0.089	6.96E-10	90.36	-0.03	8.17E-04
rs10816625	9	110,837,073	A	G	61.81	-0.079	2.21E-10	90.66	-0.11	4.20E-20
rs10760444	9	129,396,434	A	G	43.25	-0.069	1.13E-08	55.17	-0.03	9.63E-09
rs78053936	10	64,300,331	A	C	76.98	0.110	4.54E-11	98.01	0.06	4.55E-04
rs2252004	10	122,844,709	A	C	31.65	-0.070	2.63E-07	8.75	-0.03	1.72E-03
rs2248051	10	122,854,749	T	G	37.50	-0.065	5.19E-07	8.75	-0.03	1.97E-03
rs7913903	10	123,095,094	A	G	32.54	-0.067	4.31E-07	24.35	-0.03	6.19E-06
rs2912778	10	123,338,654	A	G	43.65	-0.198	5.23E-62	47.42	-0.21	1.95E-263
rs509239	11	1,885,117	A	T	23.41	0.095	6.17E-12	35.29	0.04	4.39E-10
rs1873872	11	129,473,993	A	T	65.08	-0.057	2.28E-06	47.71	-0.04	2.30E-12
rs12422552	12	14,413,931	C	G	26.98	0.068	7.47E-07	29.03	0.06	8.12E-18
rs1314084	12	28,140,277	T	C	82.54	0.136	1.84E-17	86.78	0.12	1.36E-42
rs833734	12	103,044,493	A	G	6.15	-0.103	9.02E-06	3.18	-0.08	2.49E-05
rs11067567	12	115,828,256	T	C	23.61	-0.109	8.28E-16	34.49	-0.08	1.59E-34
rs12895715	14	37,113,093	A	G	67.66	0.070	1.35E-07	78.13	0.07	6.96E-22
rs8037137	15	91,506,637	T	C	48.12	0.073	1.98E-09	86.08	0.08	1.50E-17
rs112149573	16	52,581,245	T	G	24.70	0.213	3.95E-52	24.95	0.21	1.08E-208
rs17817964	16	53,828,066	T	C	18.35	-0.075	1.35E-06	41.15	-0.06	9.09E-20
rs3893264	16	54,683,802	T	C	84.72	-0.089	9.54E-06	81.51	-0.05	9.53E-12
rs149288672	16	71,916,281	CAG	C	75.20	0.070	1.05E-06	85.98	0.04	6.89E-05
rs244373	17	53,184,949	T	C	28.67	-0.084	8.32E-10	28.43	-0.07	2.17E-27
rs2307561	18	24,503,506	A	AAGTGTT	49.40	-0.062	1.48E-06	27.04	-0.07	1.68E-21
rs10502843	18	42,378,282	A	G	14.48	-0.084	4.70E-06	6.46	-0.08	1.61E-10
rs12455117	18	42,884,026	A	T	70.34	0.094	2.55E-13	66.90	0.05	1.52E-12
rs2823126	21	16,561,704	A	G	30.75	-0.091	1.32E-10	2.19	-0.05	0.02

© 2022 Yang Y et al. *JAMA Network Open.*

rs6001335	22	39,345,966	C	G	47.22	0.064	4.14E-07	27.24	0.04	2.81E-10
rs141580207	22	40,917,540	T	TCA	75.40	-0.067	7.50E-07	91.05	-0.12	4.22E-33
**54 index SNPs in GWAS-identified loci that were not eligible for fine-mapping [b]**										
rs72906468	1	17,772,093	A	T	66.87	0.054	8.00E-05	80.02	0.03	1.82E-06
rs2992756	1	18,807,339	T	C	14.29	0.045	6.83E-03	51.19	0.05	2.67E-17
rs3790585	1	46,023,356	A	T	67.76	0.032	0.02	85.88	0.04	8.87E-07
rs11249433	1	121,280,613	A	G	97.02	-0.089	0.01	56.86	-0.10	1.21E-57
rs12710696	2	19,320,803	T	C	30.75	0.037	4.28E-03	34.19	0.04	2.68E-09
rs71801447	2	111,925,731	CTTATGTT	C	89.48	-0.051	5.04E-03	92.94	-0.06	4.23E-07
rs6762644	3	4,742,276	A	G	93.45	-0.053	0.01	64.61	-0.05	1.15E-18
rs12493607	3	30,682,939	C	G	70.63	0.039	1.68E-03	33.70	0.05	2.72E-14
rs6796502	3	46,866,866	A	G	13.79	-0.035	0.03	9.05	-0.08	1.23E-15
rs1053338	3	63,967,900	A	G	85.22	-0.036	0.03	85.29	-0.06	1.42E-11
rs58058861	3	172,285,237	A	G	32.74	0.031	0.03	17.89	0.05	2.40E-10
rs10069690	5	1,279,790	T	C	16.87	0.061	3.52E-03	27.63	0.06	1.76E-18
rs3215401	5	1,296,255	A	AG	61.61	0.035	0.02	71.17	0.07	1.13E-23
rs6555134	5	2,776,483	T	C	23.21	-0.034	0.02	56.96	-0.03	5.13E-07
rs204247	6	13,722,523	A	G	38.49	-0.043	3.63E-04	57.75	-0.05	1.56E-14
rs7765429	6	21,904,169	T	C	83.04	-0.056	9.91E-03	49.11	-0.04	1.65E-09
rs17529111	6	82,128,386	T	C	78.17	-0.034	0.02	77.73	-0.05	2.75E-10
rs17268829	7	94,113,799	T	C	71.53	-0.051	5.55E-04	71.17	-0.05	2.58E-13
rs4593472	7	130,667,121	T	C	16.27	-0.044	6.31E-03	33.20	-0.04	4.93E-11
rs144145984	8	23,644,003	CT	C	45.04	-0.041	8.60E-04	57.85	-0.03	1.46E-05
rs6472903	8	76,230,301	T	G	96.83	0.129	9.42E-05	83.60	0.08	2.48E-22
rs2849506	8	101,329,134	C	G	46.83	-0.042	4.10E-04	38.97	-0.03	1.87E-05
rs12546444	8	106,358,620	A	T	88.69	0.078	1.10E-03	90.46	0.07	1.08E-11
rs13267382	8	117,209,548	A	G	52.08	0.034	0.01	34.29	0.04	7.65E-12
rs142360995	8	118,205,719	A	G	7.64	0.077	2.08E-03	19.98	0.03	1.68E-04
rs58847541	8	124,610,166	A	G	18.75	0.033	0.04	15.31	0.06	1.91E-13
rs10759243	9	110,306,115	A	C	45.04	0.036	3.22E-03	30.82	0.06	3.08E-17
rs7904519	10	114,773,927	A	G	96.53	-0.078	0.01	50.50	-0.04	8.99E-14
rs2901157	10	119,262,365	A	G	77.08	0.055	8.46E-05	87.38	0.04	1.40E-05

© 2022 Yang Y et al. *JAMA Network Open.*

rs6597981	11	803,017	A	G	72.42	-0.031	0.02	50.20	-0.05	4.65E-14
rs10838267	11	44,368,892	A	G	29.27	0.043	1.58E-03	51.69	0.03	4.51E-08
rs1027113	12	29,140,260	A	G	75.89	0.048	7.30E-04	92.84	0.07	7.67E-12
rs78588049	12	69,180,907	A	ATTTT	17.76	-0.053	2.92E-03	20.87	-0.03	1.22E-05
rs17356907	12	96,027,759	A	G	73.61	0.046	1.08E-03	70.68	0.09	8.11E-41
rs2464195	12	121,435,475	A	G	48.02	-0.032	6.08E-03	37.77	-0.02	1.88E-03
rs9316500	13	51,094,114	T	G	35.22	0.046	1.92E-04	72.76	0.03	4.37E-06
rs2588809	14	68,660,428	T	C	2.78	0.084	0.02	18.79	0.06	1.36E-14
rs75004998	14	77,517,786	A	G	46.63	-0.036	3.31E-03	34.59	-0.03	3.40E-06
rs11627032	14	93,104,072	T	C	74.01	0.027	0.04	73.46	0.05	2.20E-11
rs8027365	15	75,808,740	A	C	65.38	0.042	6.33E-04	69.78	0.03	3.91E-07
rs2432539	16	56,420,987	A	G	38.99	0.038	2.59E-03	41.65	0.03	5.08E-07
rs4496150	16	87,085,237	A	C	45.73	-0.031	0.01	26.04	-0.04	1.25E-09
rs146699004	17	29,230,520	G	GGT	9.72	-0.058	0.04	28.63	-0.04	6.52E-09
rs745570	17	77,781,725	A	G	58.73	0.035	4.48E-03	50.50	0.04	1.56E-10
rs78269692	19	13,158,277	T	C	100.00	-1.101	0.01	95.92	-0.09	1.14E-09
rs2594714	19	13,954,571	A	G	25.69	-0.031	0.03	24.35	-0.04	2.92E-08
rs4808801	19	18,571,141	A	G	78.37	0.050	2.45E-04	65.51	0.07	6.64E-29
rs2965183	19	19,545,696	A	G	32.04	0.039	3.15E-03	33.70	0.04	3.58E-10
rs113701136	19	30,277,729	T	C	22.02	0.040	6.34E-03	28.13	0.02	2.08E-03
rs71338792	19	46,183,031	A	AT	78.57	-0.060	2.29E-04	78.23	-0.04	1.28E-08
rs12481286	20	52,287,610	T	G	32.64	0.042	2.34E-03	24.06	0.04	6.10E-09
rs9808759	21	47,780,223	T	C	18.55	0.029	0.05	7.16	0.07	5.84E-09
rs35418111	21	47,856,670	A	G	19.25	0.037	0.01	6.96	0.07	2.10E-08
rs34331122	22	19,762,428	CTT	C	57.24	-0.057	6.62E-05	43.34	-0.03	6.56E-08

[a] 66 SNPs were selected by fine-mapping at COJO-$P<10^{-5}$ and 57 of them showed consistent association patterns with breast cancer risk at $P<0.05$ in BCAC-European data. Effect sizes and *P* values of these 57 SNPs were derived from a joint analysis of all SNPs selected by fine-mapping within each loci.
[b] A total of 54 SNPs in loci that were ineligible for fine-mapping showed $P<0.05$ in our training set. Effect sizes and *P* values of these 57 SNPs were from our training set.
[c] BCAC-European data from Zhang et al. *Nat Genet*. 2020.

© 2022 Yang Y et al. *JAMA Network Open.*

eTable 6. Associations of PRS111 With Breast Cancer Risk Stratified by Age Categories in the Prospective Test Set

Age categories	OR (95% CI) [a]	*P* [a]
40-45	1.42 (1.12-1.83)	4.86E-03
45-50	1.77 (1.28-2.50)	7.58E-04
50-55	2.04 (1.42-3.05)	2.23E-04
55-60	2.14 (1.47-3.26)	1.72E-04
60-65	1.46 (1.02-2.15)	0.04
65-70	1.56 (1.06-2.34)	0.03

[a] ORs, 95% CIs and *P* values were estimated using logistic regression

© 2022 Yang Y et al. *JAMA Network Open.*

eTable 7. Association With Breast Cancer Risk for PRS111 and PRS263-meta in 10 207 Chinese Women[a]				
**Percentiles**	**PRS_{111} [b]**		**$PRS_{263\text{-}META}$ [c]**	
	**OR (95% CI) [d]**	***P* [d]**	**OR (95% CI) [d]**	***P* [d]**
<5	0.30 (0.24-0.39)	5.12E-20	0.44 (0.35-0.56)	1.33E-11
10-20	0.54 (0.43-0.67)	2.81E-08	0.51 (0.40-0.63)	4.45E-09
20-30	0.67 (0.57-0.79)	1.78E-06	0.63 (0.53-0.74)	2.36E-08
20-40	0.86 (0.75-0.98)	0.02	0.72 (0.63-0.82)	4.53E-07
40-60	1.00 (Reference)	-	1.00 (Reference)	-
60-80	1.45 (1.27-1.64)	1.61E-08	1.03 (0.91-1.16)	0.65
80-90	1.87 (1.60-2.19)	6.92E-15	1.39 (1.21-1.61)	6.30E-06
90-95	2.30 (1.88-2.80)	1.94E-16	1.54 (1.29-1.85)	2.47E-06
>95	3.39 (2.80-4.10)	1.16E-35	2.23(1.87-2.65)	1.14E-19

PRS, polygenic risk score; OR, odds ratio; CI, confidence interval; AUC, aera under the receiver operating characteristic curve.
[a] Among the whole ABCC datasets, except for those using Exome BeadChip or iCOGs as genotyping platform, 10,207 Chinese women who hx`ad both individual genetic and nongenetic data available were eligible for this analysis.
[b] PRS_{111}: the best PRS derived in the present study.
[c] $PRS_{263\text{-}META}$ was derived based on the 330-SNP European PRS reported by Zhang et al. *Nat Genet*. 2020.
[d] OR and 95% CI of each PRS percentile group compared with the reference group and P values were estimated using logistic regression.

© 2022 Yang Y et al. *JAMA Network Open.*

eReferences.

1. Shu X, Long J, Cai Q, et al. Identification of novel breast cancer susceptibility loci in meta-analyses conducted among Asian and European descendants. *Nature Communications*. 2020;11(1):1-9.
2. Lu W, Cui Y, Zheng Y, et al. Impact of newly diagnosed breast cancer on quality of life among Chinese women. *Breast Cancer Research and Treatment*. 2007;102(2):201-210.
3. Zheng W, Chow W-H, Yang G, et al. The Shanghai Women's Health Study: rationale, study design, and baseline characteristics. *Am J Epidemiol*. 2005;162(11):1123-1131.
4. Chang CC, Chow CC, Tellier LC, Vattikuti S, Purcell SM, Lee JJ. Second-generation PLINK: rising to the challenge of larger and richer datasets. *Gigascience*. 2015;4(1):s13742-015-0047-8.
5. Willer CJ, Li Y, Abecasis GR. METAL: fast and efficient meta-analysis of genomewide association scans. *Bioinformatics*. 2010;26(17):2190-2191.
6. Mavaddat N, Michailidou K, Dennis J, et al. Polygenic risk scores for prediction of breast cancer and breast cancer subtypes. *The American Journal of Human Genetics*. 2019;104(1):21-34.
7. Zhang H, Ahearn TU, Lecarpentier J, et al. Genome-wide association study identifies 32 novel breast cancer susceptibility loci from overall and subtype-specific analyses. *Nature Genetics*. 2020:1-10.
8. Zheng W, Long J, Gao Y-T, et al. Genome-wide association study identifies a new breast cancer susceptibility locus at 6q25. 1. *Nature Genetics*. 2009;41(3):324-328.
9. Long J, Cai Q, Shu X-O, et al. Identification of a functional genetic variant at 16q12. 1 for breast cancer risk: results from the Asia Breast Cancer Consortium. *PLoS Genet*. 2010;6(6):e1001002.
10. Cai Q, Long J, Lu W, et al. Genome-wide association study identifies breast cancer risk variant at 10q21. 2: results from the Asia Breast Cancer Consortium. *Human Molecular Genetics*. 2011;20(24):4991-4999.
11. Long J, Cai Q, Sung H, et al. Genome-wide association study in east Asians identifies novel susceptibility loci for breast cancer. *PLoS Genet*. 2012;8(2):e1002532.
12. Cai Q, Zhang B, Sung H, et al. Genome-wide association analysis in East Asians identifies breast cancer susceptibility loci at 1q32. 1, 5q14. 3 and 15q26. 1. *Nature Genetics*. 2014;46(8):886-890.
13. Long J, Delahanty RJ, Li G, et al. A common deletion in the APOBEC3 genes and breast cancer risk. *J Natl Cancer Inst*. 2013;105(8):573-579.
14. Han M-R, Long J, Choi J-Y, et al. Genome-wide association study in East Asians identifies two novel breast cancer susceptibility loci. *Human Molecular Genetics*. 2016;25(15):3361-3371.
15. Shu X, Long JR, Cai QY, et al. Identification of novel breast cancer susceptibility loci in meta-analyses conducted among Asian and European descendants. *Nature Communications*. Mar 5 2020;11(1)doi:10.1038/s41467-020-15046-w
16. Yang J, Ferreira T, Morris AP, et al. Conditional and joint multiple-SNP analysis of GWAS summary statistics identifies additional variants influencing complex traits. *Nature Genetics*. 2012;44(4):369-375.
17. Vilhjalmsson BJ, Yang J, Finucane HK, et al. Modeling Linkage Disequilibrium Increases Accuracy of Polygenic Risk Scores. *Am J Hum Genet*. Oct 1 2015;97(4):576-92. doi:10.1016/j.ajhg.2015.09.001
18. Ge T, Chen CY, Ni Y, Feng YA, Smoller JW. Polygenic prediction via Bayesian regression and continuous shrinkage priors. *Nat Commun*. Apr 16 2019;10(1):1776. doi:10.1038/s41467-019-09718-5
19. Prive F, Arbel J, Vilhjalmsson BJ. LDpred2: better, faster, stronger. *Bioinformatics*. Dec 16 2020;doi:10.1093/bioinformatics/btaa1029
20. Privé F, Arbel J, Vilhjálmsson BJ. LDpred2: better, faster, stronger. *Bioinformatics*. 2020.
21. Consortium IH. Integrating common and rare genetic variation in diverse human populations. *Nature*. 2010;467(7311):52.
22. Huang H, Ruan Y, Feng Y-CA, et al. Improving polygenic prediction in ancestrally diverse populations. *medRxiv* 2021
23. Zheng W, Wen W, Gao Y-T, et al. Genetic and clinical predictors for breast cancer risk assessment and stratification among Chinese women. *J Natl Cancer Inst*. 2010;102(13):972-981.
24. Robin X, Turck N, Hainard A, et al. pROC: an open-source package for R and S+ to analyze and compare ROC curves. *BMC Bioinformatics*. 2011;12(1):1-8.

© 2022 Yang Y et al. *JAMA Network Open.*
